# Underlying Mechanisms of Cooperativity, Input Specificity, and Associativity of Long-Term Potentiation Through a Positive Feedback of Local Protein Synthesis

**DOI:** 10.3389/fncom.2018.00025

**Published:** 2018-05-01

**Authors:** Lijie Hao, Zhuoqin Yang, Jinzhi Lei

**Affiliations:** ^1^School of Mathematics and Systems Science, Key Laboratory of Mathematics, Informatics and Behavioral Semantics, Ministry of Education, Beihang University, Beijing, China; ^2^Zhou Pei-Yuan Center for Applied Mathematics, MOE Key Laboratory of Bioinformatics, Tsinghua University, Beijing, China

**Keywords:** long-term potentiation, cooperativity, input specificity, associativity, local positive feedback

## Abstract

Long-term potentiation (LTP) is a specific form of activity-dependent synaptic plasticity that is a leading mechanism of learning and memory in mammals. The properties of cooperativity, input specificity, and associativity are essential for LTP; however, the underlying mechanisms are unclear. Here, based on experimentally observed phenomena, we introduce a computational model of synaptic plasticity in a pyramidal cell to explore the mechanisms responsible for the cooperativity, input specificity, and associativity of LTP. The model is based on molecular processes involved in synaptic plasticity and integrates gene expression involved in the regulation of neuronal activity. In the model, we introduce a local positive feedback loop of protein synthesis at each synapse, which is essential for bimodal response and synapse specificity. Bifurcation analysis of the local positive feedback loop of brain-derived neurotrophic factor (BDNF) signaling illustrates the existence of bistability, which is the basis of LTP induction. The local bifurcation diagram provides guidance for the realization of LTP, and the projection of whole system trajectories onto the two-parameter bifurcation diagram confirms the predictions obtained from bifurcation analysis. Moreover, model analysis shows that pre- and postsynaptic components are required to achieve the three properties of LTP. This study provides insights into the mechanisms underlying the cooperativity, input specificity, and associativity of LTP, and the further construction of neural networks for learning and memory.

## 1. Introduction

Learning and memory are fundamental mental processes that are critical for adaptation and survival (Kandel et al., 2014; Alberini and Kandel, 2015). In neuroscience, one of the most fascinating questions is to understand how the brain stores information and provides a proper response to suitable stimuli. The cellular changes that underlie memory storage are thought to associate with synaptic plasticity (Martin et al., 2000; Nicoll, 2017).

Synaptic plasticity is the ability of synaptic connections to change over time (Purves et al., 2004; Byrne and Roberts, 2009). The plasticity can last for either a short term of < 30 min or a long term that can persist many hours (Byrne and Roberts, 2009). Long-lasting forms of synaptic plasticity, either long-term potentiation (LTP) or long-term depression (LTD), are the cellular bases of learning and memory (Bliss and Collingridge, 1993; Ito, 2002; Malinow and Malenka, 2002; Whitlock et al., 2006). LTP is a process whereby brief periods of synaptic activity can produce a long-lasting increase in synapse strength. There are three properties of LTP: (1) cooperativity, (2) input specificity, and (3) associativity, which are essential for learning and memory in mammals (Kitajima and Hara, 1991; Kandel et al., 2013). LTP induction requires the cooperative interaction of afferent fibers in certain systems, such as the Schaffer collateral pathway (Gustafsson et al., 1987; Ballyk and Goh, 1992; Jung and Larson, 1994; Byrne and Roberts, 2009; Kandel et al., 2013). LTP is input specific; it is restricted to synapses activated by a strong stimulation rather than all synapses that contact the same neuron (Bliss and Collingridge, 1993; Frey and Morris, 1997; Nishiyama et al., 2000; Tao et al., 2001; Purves et al., 2004; Kandel et al., 2013). The associativity is important for LTP, by which a synapse being not produce LTP with weak input can undergo LTP when the synapse is coactivated via a strong input (Barrionuevo and Brown, 1983; Kelso and Brown, 1986; Bliss and Collingridge, 1993; Humeau et al., 2003; Purves et al., 2004; Kandel et al., 2013). In learning and memory, the cooperativity of LTP indicates that only events that trigger sufficient inputs can result in memory storage, input specificity ensures the accuracy of memory storage, and associativity is a mechanism of associative learning (Blair et al., 2001; Kandel et al., 2013).

Long-lasting forms of synaptic plasticity are closely associated with gene expression and protein synthesis at synapse. Protein synthesis plays key roles in the modulation of long-term synaptic plasticity and the consolidation of memory (Kelleher et al., 2004; Takei et al., 2004; Sutton and Schuman, 2006; Helmstetter et al., 2008; Tanaka et al., 2008). Translation machinery and mRNAs are found locally in dendrites and even within synaptic spines, and local protein synthesis is required for long-term plasticity (Steward and Levy, 1982; Steward and Reeves, 1988; Kang and Schuman, 1996; Steward and Schuman, 2001; Ostroff et al., 2002; Sutton et al., 2004; Sutton and Schuman, 2005, 2006; Sutton et al., 2006). Activation of mammalian target of rapamycin (mTOR) at the synaptic region can initiate local translation of proteins, including brain-derived neurotrophic factor (BDNF), which is crucial for synaptic plasticity (Tang et al., 2002; Takei et al., 2004; Besse and Ephrussi, 2008; Tanaka et al., 2008; Hoeffer and Klann, 2009; Fortin et al., 2012; Park and Poo, 2013; Harward et al., 2016; Hedrick et al., 2016). BDNF mRNAs are accumulated and locally translated in dendrites (Park and Poo, 2013; Harward et al., 2016; Hedrick et al., 2016), and the translation products are secreted into the clefts; the secretion of BDNF vesicles is dependent on the intracellular calcium level (Lessmann et al., 2003; Kolarow et al., 2007; Wonga et al., 2015). Secreted BDNF can in turn induce mTOR-dependent local activation of the translation machinery and lead to local protein synthesis in the dendrites (Takei et al., 2004; Hoeffer and Klann, 2009; Fortin et al., 2012; Park and Poo, 2013).

Many computational models have been developed in order to understand synaptic plasticity and to investigate the possible mechanisms associated with learning and memory (Lisman, 1989; Holmes and Levy, 1990; Kitajima and Hara, 1991; Migliore et al., 1997; Migliore and Lansky, 1999; Lisman and Zhabotinsky, 2001; Shouval et al., 2002; Migliore et al., 2015). In these models, Ca^2+^/calmodulin-dependent signals play an important role in synaptic plasticity, and the calcium flux is often mediated by postsynaptic N-methyl-D-aspartate (NMDA) channels (Lisman, 1989; Holmes and Levy, 1990; Lisman and Zhabotinsky, 2001; Shouval et al., 2002). In addition to the mechanisms of LTP induction, the main properties of LTP are also discussed, separately, using different models. Kitajima and Hara (1991) introduced a model for the cooperativity and associativity of LTP in the hippocampus through the spread of synaptic potentials. A model was proposed by Migliore et al. (1997) to interpret the experiments in terms of molecular processes that might be involved in associative memory. The model suggests that retrograde messengers could have a critical role in the induction and maintenance of associative LTP. These existing models did not consider local protein synthesis that is crucial for synaptic plasticity. It is not known how gene transcription and local protein synthesis are integrated with electrical activity of a neuron to mediate the formation of LTP, in particular, how the three key properties of LTP can be modeled with a unified model framework.

In this paper, we introduce a computational model based on the molecular processes involved in synaptic plasticity, and employ the model to investigate the underlying mechanisms of the three properties of LTP: cooperativity, input specificity, and associativity. The model integrates the regulation of gene expression with the membrane action potential of a hippocampal pyramidal neuron. In the model, we introduce the hypothesis of a local positive feedback loop that involves postsynaptic protein synthesis and secretion at each synapse. The model is able to reproduce the cooperativity, input specificity, and associativity of LTP under suitable parameter regions. Bifurcation analysis based on the simple motif of the positive feedback loop illustrates the existence of bistability of the synaptic response due to the positive feedback motif, which is the basis of LTP induction. Moreover, the concentrations of postsynaptic calcium and BDNF mRNA before and after stimulation are in accordance with the values predicted by the bifurcation analysis of the local positive feedback loop, which indicates that the local motif is essential for the behavior of synaptic plasticity. Based on the model simulations, we study the role of the pre- and postsynaptic components in the realization of LTP; the results show that both presynaptic neurotransmitter release and postsynaptic depolarization are required for the induction of LTP. Furthermore, we discuss the effects of the number of active synapses in the input pathway and the coupling conductance between the soma and spine in cooperativity and associativity, respectively. The proposed model provides insights into the underlying mechanisms of cooperativity, input specificity, and associativity of LTP, and provides a module that may be used to build neural networks.

## 2. Model and method

### 2.1. Model description

**Figure 1:**
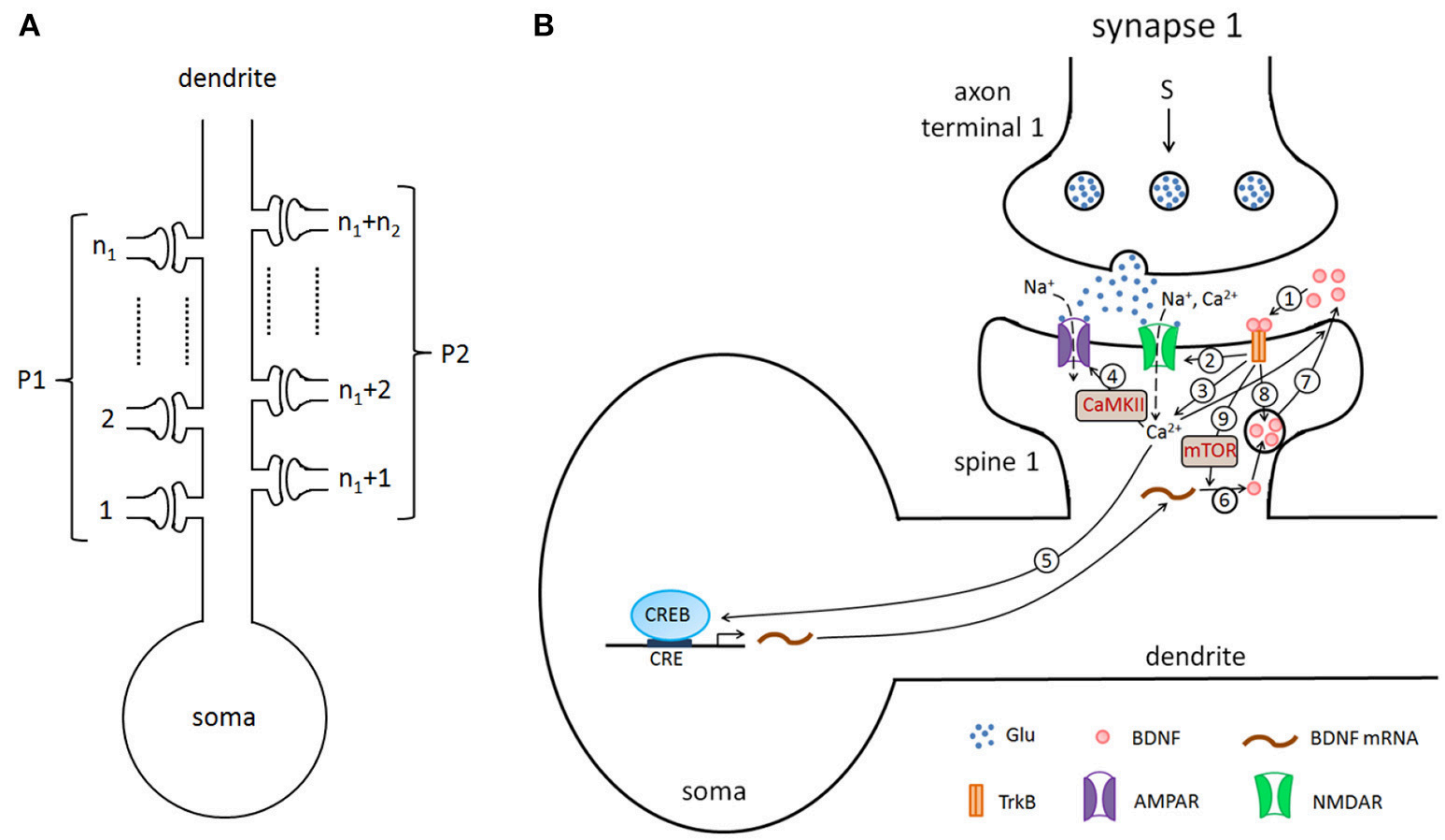
**(A)** The pyramidal cell model with *n* excitatory synapses. The synapses are divided into two type pathways, P1 (*n*_1_ synapses) and P2 (*n*_2_ synapses), which deliver different stimuli to induce LTP. **(B)** Detailed illustration of the biological processes in the model. Here, we showed the processes from the presynaptic side to the synaptic cleft and then to the postsynaptic side, taking synapse 1 as an example. The presynaptic stimulation causes the release of the neurotransmitter Glu (blue dots) from the presynaptic nerve terminal into the cleft. The neurotransmitter promotes the opening of postsynaptic AMPA and NMDA glutamate receptor channels and results in Na^+^ and Ca^2+^ influx. BDNF (pink circles) binds to the postsynaptic TrkB receptor (1), increases the conductance of the NMDA channel (2), increases the postsynaptic Ca^2+^ concentration (3), subsequently regulates AMPA channel conductance via phosphorylation of CaMKII (4), and activates CREB proteins to induce BDNF transcription (5). BDNF transcription can be accumulated and locally translated into proteins in dendrites (6), and proteins are secreted from dendritic spines (7). Binding of secreted BDNF to the postsynaptic TrkB receptor leads to endocytic uptake of BDNF-TrkB complexes (8). BDNF in the synaptic cleft can induce postsynaptic mTOR-dependent local translation of BDNF mRNAs (9), and the synthesized BDNF proteins in turn are secreted into the cleft to form a local positive feedback loop.

In this study, we considered a hippocampal pyramidal neuron (PN) model with *n* excitatory synapses (see Figure 1A). In model simulations, to generate different properties of LTP, the synapses were divided into two type pathways, P1 (*n*_1_ synapses) and P2 (*n*_2_ synapses) (here *n*_1_+*n*_2_ = *n*), which can deliver different stimuli. Each synapse consists of three components: presynaptic axon terminal, synaptic cleft, and spine on the postsynaptic cell. Figure 1B shows all biochemical processes at a synapse from the presynaptic to the synaptic cleft and then to the postsynaptic (detailed below). A major assumption of the model is that BDNF in the synaptic cleft can induce postsynaptic mammalian target of rapamycin (mTOR)-dependent local translation of BDNF mRNAs, and the synthesized BDNF proteins in turn are secreted into the cleft to form a positive feedback loop (Figure 1B).

Synaptic vesicles clustered in the presynaptic nerve terminal are filled with neurotransmitter that can be released into the cleft via presynaptic stimulations (Figure 1B). Released neurotransmitters can promote the opening of postsynaptic α-amino-3-hydroxy-5-methyl-4-isoxazolepropionic acid (AMPA) and N-methyl-D-aspartate (NMDA) glutamate receptor channels and elicit excitatory postsynaptic currents. Meanwhile, the postsynaptic Ca^2+^ increase due to the influx through NMDA receptors (NMDARs) to trigger downstream signaling pathways (Appendix, Equation A24 in Supplementary Material).

An increase in the postsynaptic calcium levels leads to the autophosphorylation of Ca^2+^/calmodulin (Cam)-dependent protein kinase II (CaMKII), which in turn phosphorylates the AMPA receptor (AMPAR) glutamate receptor 1 (GluR1) subunit and produces the GluR1 insertion, resulting in an enhancement of AMPAR-mediated transmission and synaptic strength at the activated synapse (Hayashi et al., 2000; Malinow and Malenka, 2002; Bredt and Nicoll, 2003; Lisman et al., 2012). AMPARs also mediate the postsynaptic depolarization; the number of AMPARs at synapses determines the dynamics of fast glutamatergic signaling (Shi et al., 1999; Bredt and Nicoll, 2003; Gan et al., 2015). The reduction of synaptic efficacy through dephosphorylation and internalization of AMPARs is governed by the Ca^2+^-dependent activation of a postsynaptic protein phosphatase cascade that involves protein phosphatase-1 (PP1) (Lisman, 1989; Yan et al., 1999; Philpot and Bear, 2002; Kennedy, 2016). Thus, postsynaptic Ca^2+^ can either increase or decrease AMPA channel conductance (Appendix, Equation A15 in Supplementary Material).

The postsynaptic Ca^2+^ signaling can be enhanced by the tropomyosin-related kinase B (TrkB) receptor in the postsynaptic cell membrane. The phosphorylation of TrkB receptors are activated by binding to BDNF in the cleft (pink circles in Figure 1). Activated TrkB receptors can result in the release of Ca^2+^ from internal calcium stores through phospholipase C-γ (PLC-γ) (Tyler et al., 2002; Park and Poo, 2013), and upregulate the Ca^2+^ influx by increasing of the NMDA channel conductance (Tyler et al., 2002; Black, 1999). These processes are described by Equatios A16, A24, andA25 in the Appendix (Supplementary Material).

The phosphorylated TrkB receptors also lead to Ras-dependent activation of the mitogen-activated protein kinase (MAPK) extracellular signal-regulated kinase (ERK) (Finkbeiner et al., 1997). Both MAPK and CaMKII signaling cascades converge to a critical transcription factor, cyclic AMP response element (CRE)-binding protein (CREB), to induce the phosphorylation of CREB and trigger gene transcription (Finkbeiner et al., 1997; Tyler et al., 2002; Ying et al., 2002; Thomas and Huganir, 2004; Benito and Barco, 2010). The BDNF promoter IV contains the CREB binding site CRE, and activated CREB can promote the transcription of BDNF in the postsynaptic neuron (Martinowich et al., 2003; Park and Poo, 2013). BDNF transcripts can be accumulated and locally translated at dendrites; BDNF proteins are secreted from dendritic spines (Park and Poo, 2013; Harward et al., 2016; Hedrick et al., 2016), and the secretion of BDNF vesicles is dependent on the intracellular calcium level (Lessmann et al., 2003; Kolarow et al., 2007; Wonga et al., 2015). In the postsynaptic dendritic spines, the BDNF protein can induce mTOR-dependent local activation of the translation machinery, and this activation leads to dendritic synthesis of proteins, including BDNF (Takei et al., 2004; Hoeffer and Klann, 2009; Fortin et al., 2012; Park and Poo, 2013). These processes of BDNF production are formulated with Equations A17–A23 in the Appendix (Supplementary Material). Furthermore, BDNF regulates the expression of AMPA receptor subunits in hippocampal neurons and induces the delivery of AMPA receptors to the synapse (Appendix, Equation A15 in Supplementary Material) (Caldeira et al., 2007; Slipczuk et al., 2009; Li and Wolf, 2011; Musumeci and Minichiello, 2011). Synaptic activation can result in the exocytosis of both endogenous BDNF-containing post-Golgi granules and endosomes containing endocytosed BDNF (Wonga et al., 2015). In our model, we consider the secretion of BDNF from the postsynaptic dendrite and the endocytic uptake of BDNF into the postsynaptic dendritic spines, although these processes may also occur presynaptically.

The model formulations are detailed in the Appendix (Supplementary Material). In the model formulation, the postsynaptic membrane potential is described base on the Morris-Lecar (ML) model (Morris and Lecar, 1981), with the combination of vesicle release from the synapse triggered by different protocol stimuli. In the formulation, changes in the glutamate concentration in the cleft at each synapse upon the stimulation of vesicle release is represented by the delta function (Tsodyks and Markram, 1997; Nadkarni et al., 2008). The cell membrane potential of the multi-compartment pyramidal cell model is adapted from the ML model (Morris and Lecar, 1981). The spines include AMPA and NMDA channels; changes in the maximal conductance of both AMPA and NMDA channels are formulated as Michaelis-Menten functions (Alon, 2006). Postsynaptic BDNF transcription is dependent on CREB, and BDNF translation is dependent on the level of BDNF in the synaptic cleft; both transcription and translation rates are given by Hill type functions (Alon, 2006).

### 2.2. Methods

In model simulations, we designed stimulus protocols and numerically solved the differential equations. Based on model simulations, LTP is measured by the increases and persistence of the excitatory postsynaptic potential (EPSP) and excitatory postsynaptic current (EPSC).

**Figure 2:**
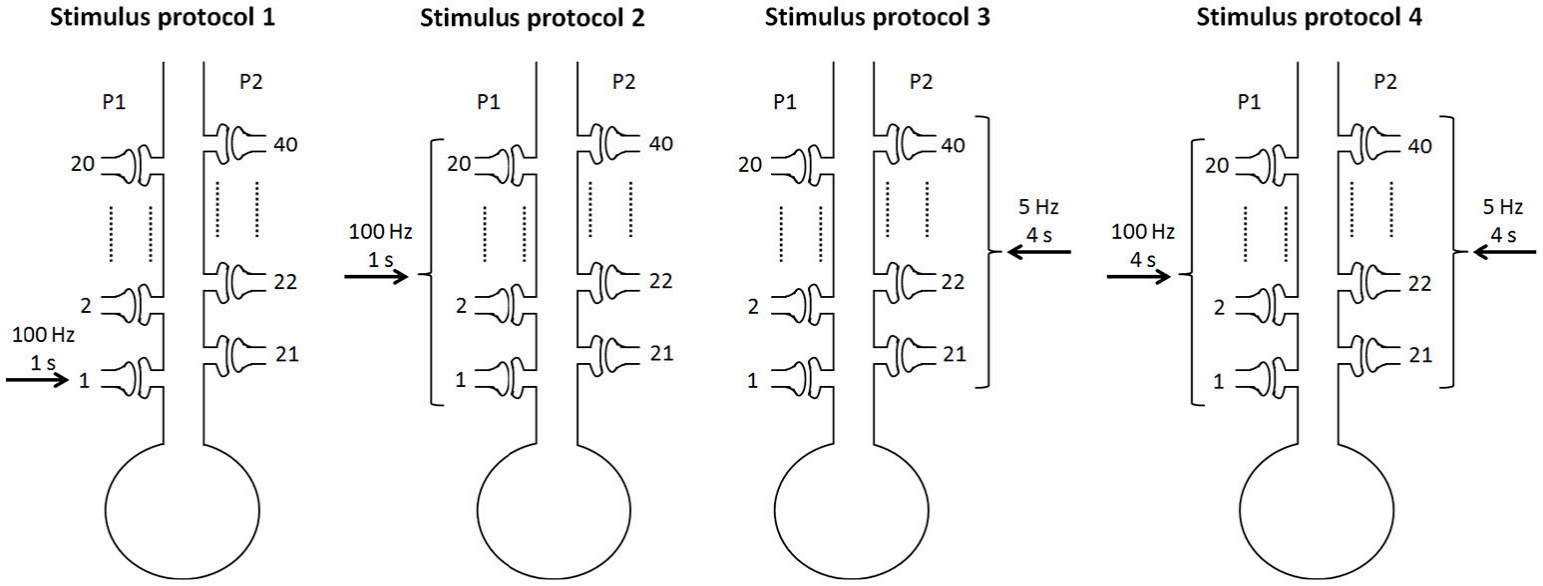
Four stimulus protocols. Stimulus protocol 1: synapse 1 in the P1 pathway receives a stimulus of 100 Hz for 1 s. Stimulus protocol 2: all synapses in the P1 pathway receive a stimulus of 100 Hz for 1 s. Stimulus protocol 3: all synapses in the P2 pathway receive a weak stimulus (5 Hz for 4 s). Stimulus protocol 4: all synapses in the P1 pathway receive a strong stimulus (100 Hz for 4 s) and all synapses in the P2 pathway receive a weak stimulus (5 Hz for 4 s) simultaneously.

To consider different stimulus protocols to reveal the cooperativity, input specificity, and associativity of LTP, we divided the synapse into two pathways, the P1 pathway with *n*_1_ synapses and the P2 pathway with *n*_2_ synapses, and applied different protocols to the two pathway synapses. In simulations, we assumed *n* = 40 and *n*_1_ = *n*_2_ = 20 when not noted specifically. Experimentally, the persistence of BDNF-TrkB signaling and the contribution to LTP induction and maintenance is highly sensitive to the pattern of synaptic stimulations (Panja and Bramham, 2014). We adopted the 100 Hz tetanus that is widely used to achieve the three important properties of LTP. To achieve this goal, four stimulus protocols were designed in our simulations (Figure 2):

only one synapse (synapse 1) in the P1 pathway receives a stimulus of 100 Hz for 1 s;
all synapses in the P1 pathway receive a stimulus of 100 Hz for 1 s;
all synapses in the P2 pathway receive a weak stimulus of 5 Hz for 4 s; synapses in the P1 pathway receive a strong stimulus of 100 Hz for 4 s, and all synapses in the P2 pathway receive a weak stimulus of 5 Hz for 4 s.

Numerical simulations were performed with MATLAB. The differential equations were solved using the Euler scheme with a time step of 0.01 ms. The injection of a short current pulse into the presynaptic terminal elicits a single action potential (Destexhe et al., 1998). The depolarization of the action potential activates high-threshold calcium channels and produces a rapid influx of calcium, resulting in a pulse of transmitter release when an action potential arrives at the presynaptic terminal (Destexhe et al., 1998). Thus, in simulations, a stimulus with the frequency of 100 Hz indicated that the frequency of the presynaptic vesicle release in response to the stimulation was 100 Hz. The amplitudes of EPSPs and EPSCs before and after the stimulations were computed to test the synaptic strength and determine whether an input pathway was potentiated. In the following, EPSP and EPSC in P1 or P2 were evoked by a single stimulus, i.e., a single vesicle release at synapse 1 or synapse 21, respectively.

Bifurcation analyses in the current study were performed with XPPAUT (Ermentrout, 2003).

## 3. Results

### 3.1. Properties of LTP based on various stimulus protocols

**Figure 3:**
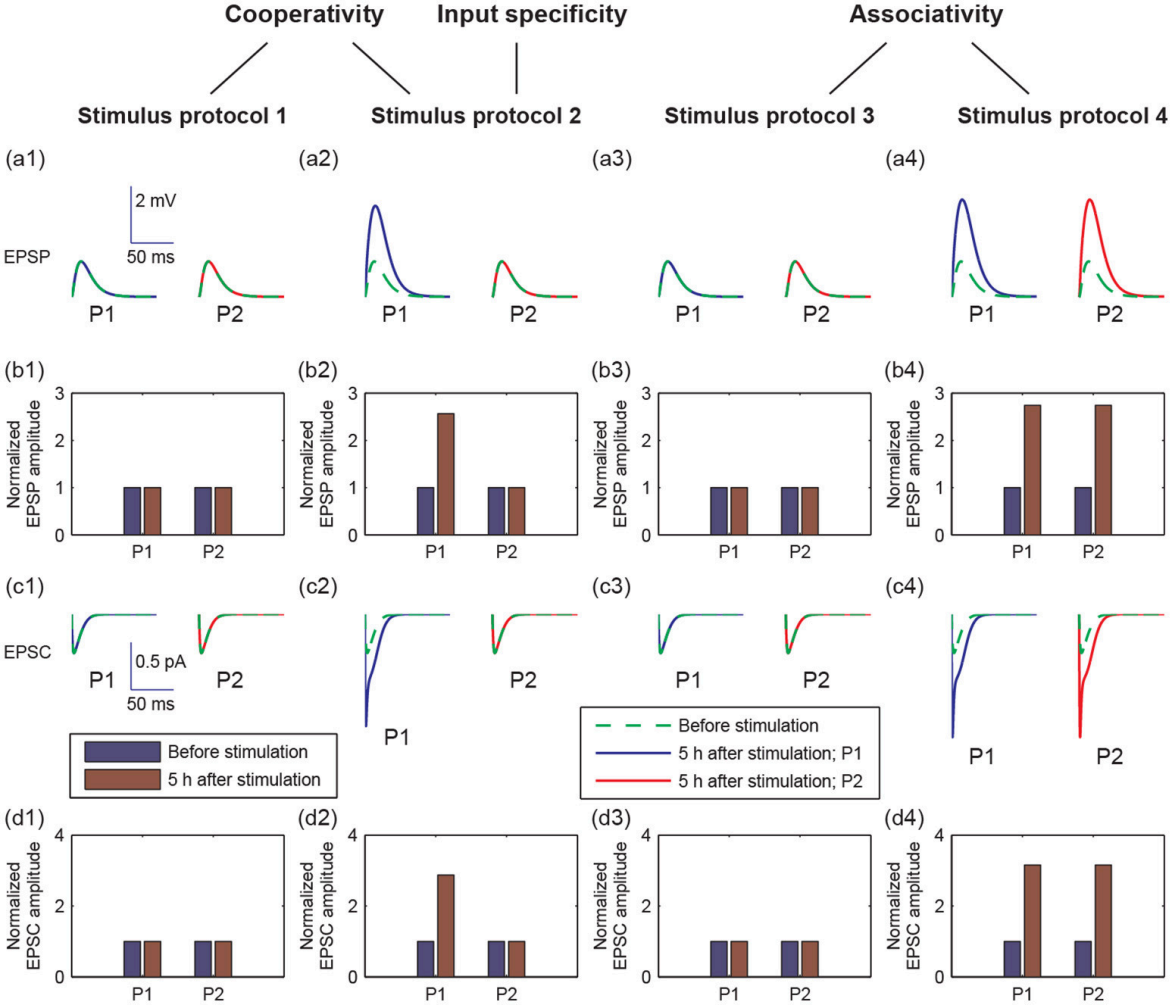
Different postsynaptic responses induced by the four stimulus protocols. **(a1–a4)** Representative excitatory postsynaptic potential (EPSP) traces before stimulation (green dashed lines) and at 5 h after stimulation (blue or red solid lines). **(b1–b4)** Amplitudes of EPSP, normalized to the EPSP amplitude without stimulation, before (blue bars) and at 5 h after stimulation (brown bars). **(c1–c4)** Representative AMPA-mediated excitatory postsynaptic current (EPSC) traces before stimulation (green dashed lines) and at 5 h after stimulation (blue or red solid lines). **(d1–d4)** AMPA-mediated EPSC amplitudes, normalized to the AMPA-mediated EPSC amplitude without stimulation, before (blue bars) and at 5 h after stimulation (brown bars). In all figures, values of P1 are represented by that at synapse 1, and values of P2 are represented by that at synapse 21, respectively. In **(a,c)**, x-axes are the time from a given time point (before stimulation or 5 h after stimulation), y-axes are EPSP or EPSC. Scales of the axes are given by insets in **(a1** or **c1)**, respectively.

LTP has three important properties: cooperativity, input specificity, and associativity (Kandel et al., 2013). Cooperativity means the property of nearly simultaneous activation of a large number of afferent axons to induce LTP. The input specificity is manifested at synapses of active, but not inactive, presynaptic afferents to the postsynaptic cell. Associativity means that coactivation of weak inputs with strong inputs onto the same neuron can strengthen the weak inputs. Since LTP was reflected by increases in the size of the excitatory postsynaptic potential (EPSP) and excitatory postsynaptic current (EPSC), we examined the properties of LTP by EPSPs and AMPA-mediated EPSCs in pathways P1 and P2 in response to the four stimulus protocols (Figure 3).

To examine cooperativity, we induced a 100 Hz stimulus to synapse 1 (protocol 1), and there was no change in the amplitudes of EPSP and AMPA-mediated EPSC in synapse 1 at 5 h after simulation (Figures 3a1–d1). Next, we induced 100 Hz stimuli to all synapses in the P1 pathway (protocol 2), and the amplitudes of EPSP and AMPA-mediated EPSC clearly increased in the P1 pathway (Figures 3a2–d2). These results reveal that the cooperativity of LTP induction potentiated by the synaptic transmission in P1 can be induced when many synapses are simultaneously activated.

Moreover, when we applied the protocol 2, the potentiation of synaptic transmission only occurred in P1 and not in P2. This result reveals that the input specificity of LTP is only induced by the strong stimulation in P1.

To test the associativity, we first applied a weak stimulus to the P2 pathway (protocol 3), and found that neither P1 nor P2 synapses showed changes in EPSP and EPSC amplitudes (Figures 3a3–d3). Next, we applied a weak stimulus to the P2 pathway paired with a strong stimulus to the P1 pathway (protocol 4). Both amplitudes of EPSP and EPSC increase significantly in both P1 and P2 synapses at 5 h after simulation (see Figures 3a4–d4). These results showed that associative LTP occurs when a weak stimulus to P2 is strengthened by the strong stimulus to P1.

**Figure 4:**
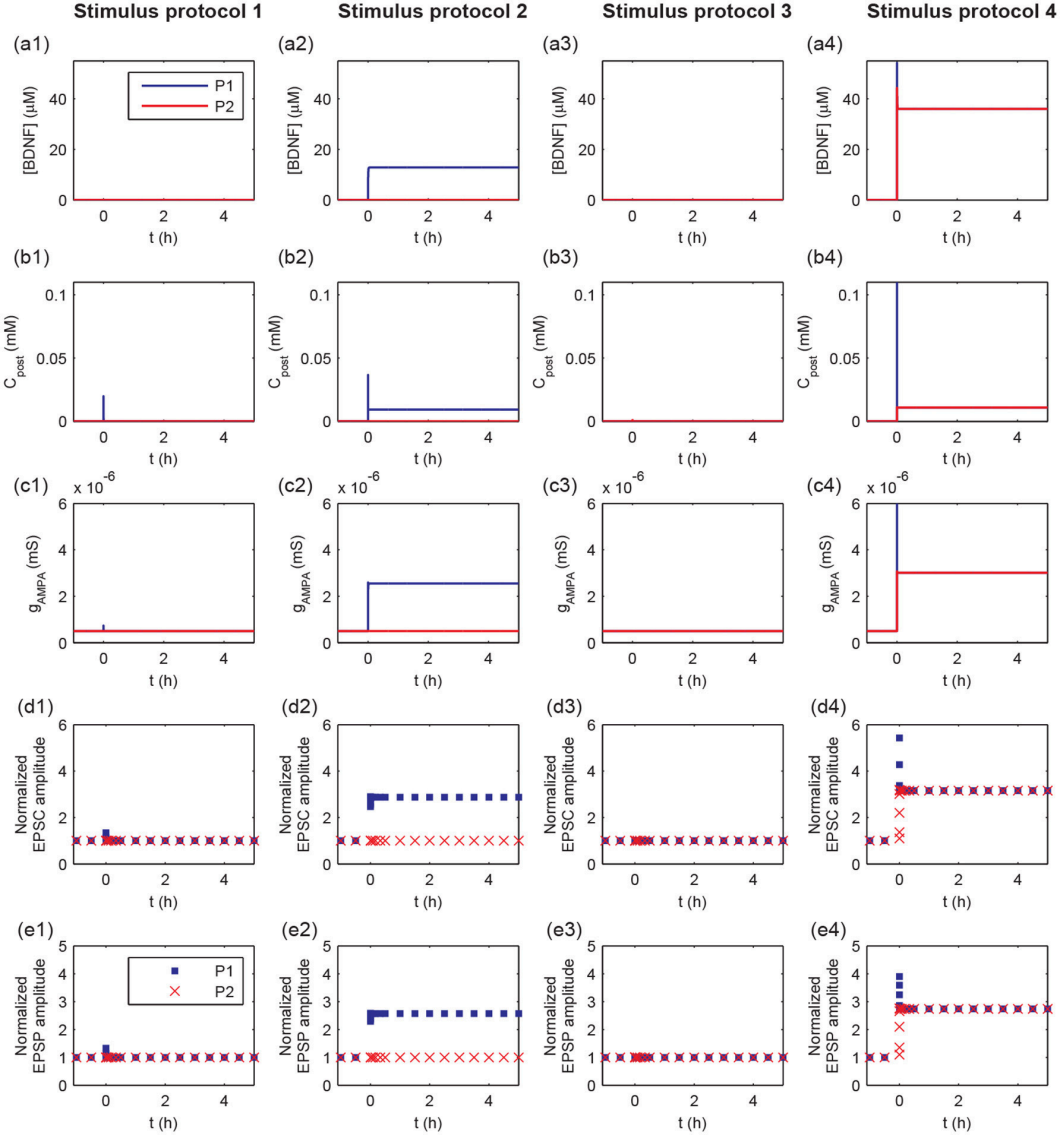
Time courses of cleft BDNF concentrations [BDNF] **(a1–a4)**, the postsynaptic Ca^2+^ concentrations (*C*_post_) **(b1–b4)**, AMPA channel maximal conductances (*g*_AMPA_) **(c1–c4)**, normalized AMPA-mediated EPSC amplitudes **(d1–d4)**, and normalized EPSP amplitudes **(e1–e4)** in P1 and P2 before and after stimulation (four stimulus protocols). In all protocols, the stimulations are given at *t* = 0.

To further explore the detailed responses of postsynaptic behavior, we examined the dynamics of [BDNF], postsynaptic Ca^2+^, AMPA conductance, and EPSC and EPSP amplitudes after stimulus (Figure 4). Activation of a single synapse (protocol 1) only resulted in an increased pulse of postsynaptic Ca^2+^ (Figure 4b1), but it was not able to induce the high level P1 cleft BDNF concentration [BDNF] (Figure 4a1). Activation of multiple synapses (protocol 2) triggered an increased pulse in postsynaptic Ca^2+^, leading to the Ca^2+^ release from internal calcium stores and eventually a high postsynaptic Ca^2+^ concentration after stimulation (Figure 4b2, blue line). Thus, the P1 cleft BDNF concentration increased and was maintained at a high level after stimulation (Figure 4a2, blue line). The high level of postsynaptic Ca^2+^ (*C*_post_) led to the elevated AMPA channel maximal conductance in P1 (Figure 4c2), which resulted in persistent increases in the amplitudes of AMPA-mediated EPSC and EPSP (Figures 4d2,e2). Consequently, the realization of the cooperativity of LTP was attributed to the activation of many synapses in P1 that could induce the transition of EPSC and EPSP amplitudes from low to high. Nevertheless, the cleft BDNF concentration in P2 remained low after the protocol 2 stimulus (Figure 4a2, red line), resulting in low postsynaptic Ca^2+^ concentrations and low postsynaptic AMPA channel maximal conductances at synapses in P2 (Figures 4b2–c2). Therefore, strong activity could only cause LTP at active synapses in P1 and could not induce LTP at inactive synapses in P2, demonstrating the input specificity of LTP.

The weak stimulus in P2 had no effect on the concentrations of the cleft BDNF, postsynaptic Ca^2+^, and maximal conductance of AMPA receptor channels in both pathways (see Figures 4a3–c3). However, when the weak stimulus in P2 was paired with the strong stimulus in P1, the concentrations of cleft BDNF, postsynaptic Ca^2+^, and maximal conductance of AMPA receptor channels in both pathways increased and persisted at high levels after stimulation (see Figures 4a4–c4). Hence, the combination of a strong stimulus in P1 with a weak stimulus in P2 led to obvious differences in the amplitudes of AMPA-mediated EPSC and EPSP in the two pathways, as compared with a weak stimulus in P2 (see Figures 4d3,e3,d4, and e4). These results illustrate the associativity of LTP.

Taken together, the results in Figures 3, 4 showed that the four stimulus protocols were able to demonstrate the three important properties of LTP. Protocols 1 and 2 together showed the cooperativity of LTP, in which the activation of a single synapse could not induce LTP, which required the cooperative interaction of many synapses in P1. Protocol 2 also indicated the input specificity of LTP, in which the strong stimulus in P1 could give rise to long-term modification of the excitatory synapses in P1 without initiating LTP at inactive synapses in P2. Protocols 3 and 4 illustrated the associative property of LTP, in which the weak stimulus in P2 alone could not trigger LTP, while synapses in both pathways were strengthened when the weak stimulus in P2 was paired with strong stimulus in P1.

### 3.2. Underlying mechanism of LTP using a toy model with a local positive feedback loop

#### 3.2.1. Bistability of a local positive feedback loop of BDNF production

**Figure 5:**
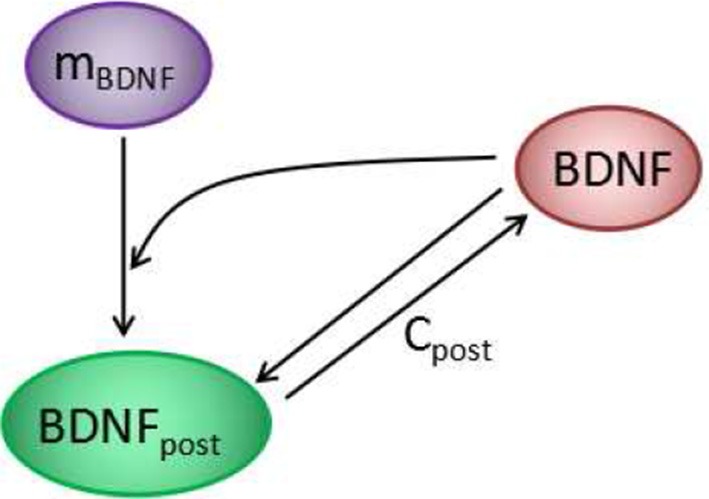
The positive feedback loop at each synapse of the model in Figure 1. Cleft BDNF promotes the translation of BDNF mRNA (m_BDNF_) in dendritic spines by binding to TrkB receptors, and the synthesized postsynaptic BDNF (BDNF_post_) proteins can be secreted into the synaptic cleft to form a positive feedback loop.

In experiments, a transient strong stimulation can lead to a long-lasting increase in the strength of synaptic connections (Bliss and Lømo, 1973; Morgan and Teyler, 2001). The transition of synaptic efficacy from low to high after stimulation indicated a bimodal response in the synapse. In the proposed model, the concentration of cleft BDNF is crucial for AMPA and NMDA channel conductance and, hence, for the increasing of postsynaptic potential. To obtain a simple model for the underlying mechanism of LTP induction, we analyzed the motif of BDNF induction, which includes the local positive feedback loop of postsynaptic protein synthesis and secretion (Figure 5). In this motif, cleft BDNF promotes the translation of BDNF mRNA in the dendritic spine by binding to TrkB receptors, and the synthesized postsynaptic BDNF proteins can be secreted into the synaptic cleft to form a positive feedback loop. This motif can be represented as a toy model by second-order differential Equations 1, 2 derived from Equations A19, A20 in the Appendix (Supplementary Material).

(1) $$\begin{array}{l} \frac{d{\left[\text{BDNF}\right]}_{\text{post}}}{dt}=k_{1}m_{\text{BDNF}}+k_{2}m_{\text{BDNF}}\frac{{\left[\text{BDNF}\right]}^{2}}{K_{2}^{2}+{\left[\text{BDNF}\right]}^{2}}\\ \;+k_{\text{in}}[\text{BDNF}]-k_{\text{out}}C_{\text{post}}{\left[\text{BDNF}\right]}_{\text{post}}\\ \;-k_{dB}{\left[\text{BDNF}\right]}_{\text{post}}, \end{array}$$

(2) $$\begin{array}{l} \frac{d[\text{BDNF}]}{dt}=k_{\text{out}}C_{\text{post}}{\left[\text{BDNF}\right]}_{\text{post}}-k_{\text{in}}[\text{BDNF}]\\ \;-k_{dB}[\text{BDNF}]. \end{array}$$

In Equations (1, 2), the postsynaptic Ca^2+^ (*C*_post_) and BDNF mRNA (*m*_BDNF_) are adjustable parameters.

**Figure 6:**
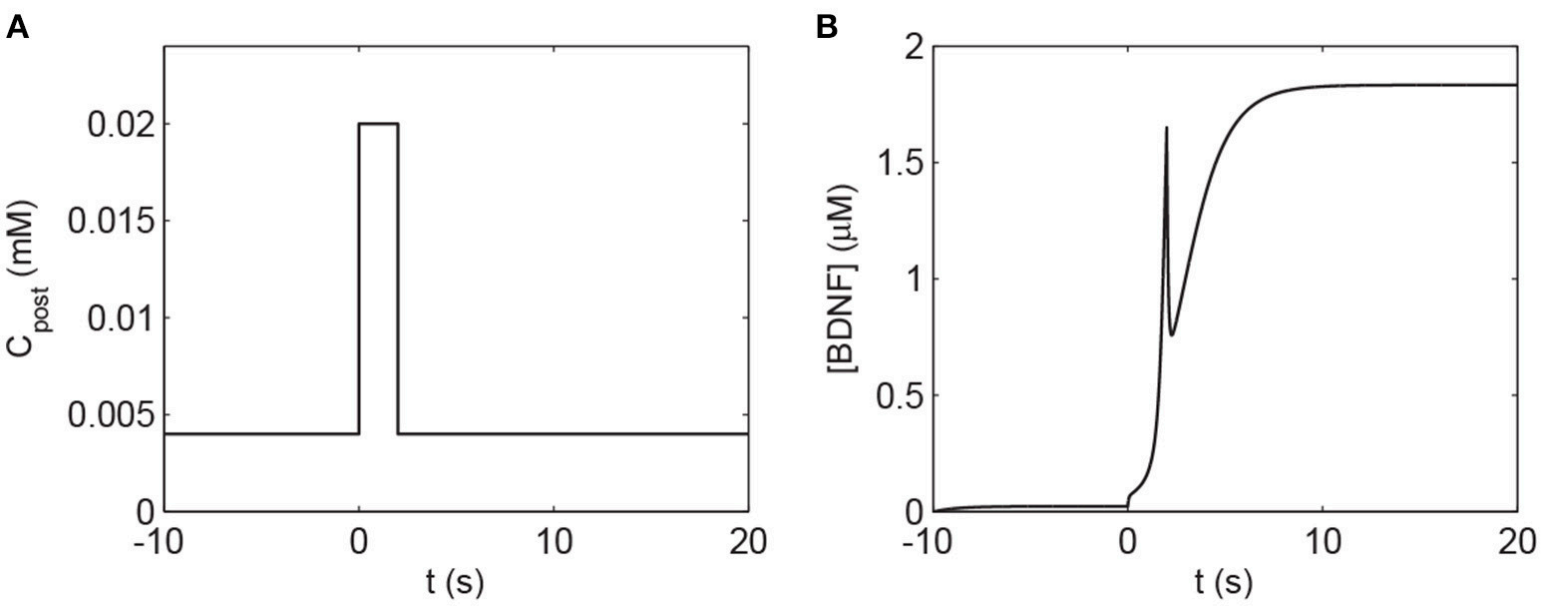
The transient increase in *C*_post_ induces a high level of [BDNF] according to Equations (1), (2). **(A)** The 2 s elevation of *C*_post_ to 0.02 mM. **(B)** The corresponding [BDNF] time course. The basal value of *C*_post_ is assumed to be 0.004 mM, and the parameter *m*_BDNF_ = 0.1681 μM. Other parameter values are presented in Table A1.

**Figure 7:**
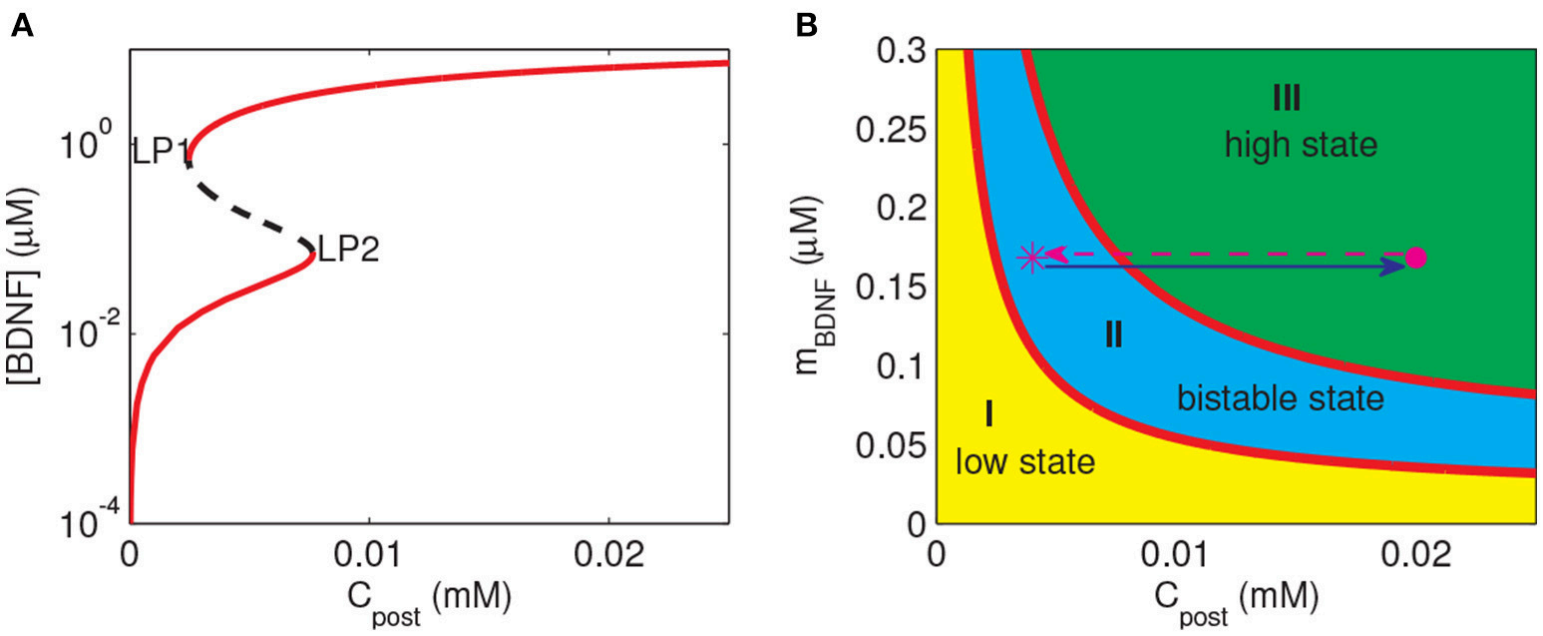
Bifurcation diagrams of the simple motif model. **(A)** Bifurcation diagram of [BDNF] vs. *C*_post_, where *m*_BDNF_ = 0.1681 μM. Limit points (fold bifurcation points) are marked as LP1 and LP2. **(B)** Two-parameter bifurcation diagram in the (*C*_post_, *m*_BDNF_) plane. The magenta star and dot depict the positions of (*C*_post_, *m*_BDNF_) at the basal level of *C*_post_ and during the *C*_post_ elevation, respectively.

To investigate the response of the BDNF concentration with different values of *C*_post_ and *m*_BDNF_, we considered *m*_BDNF_ = 0.1681 μM as the level prior to the stimulus, and varied *C*_post_. We induced a 2-s increase in *C*_post_ from 0.004 mM to 0.02 mM, [BDNF] transited from a low to a high state and then remained in the high level state even when *C*_post_ returned to its basal level, demonstrating bistable responses (Figure 6). We further confirmed this bistability through a bifurcation analysis with respect to *C*_post_ and *m*_BDNF_ (Figure 7). We fixed *m*_BDNF_ and increased *C*_post_, which resulted in a transition from one stable steady state (low-level [BDNF]) to the coexistence of two stable steady states (low and high-level [BDNF]) along with an unstable state via a fold bifurcation (LP1), and further to only one stable steady state (high-level [BDNF]) via another fold bifurcation (LP2) (Figure 7A). We performed a two-parameter bifurcation analysis, and the parameter plane (*C*_post_, *m*_BDNF_) could be divided into three regions by two fold bifurcation curves (Figure 7B); bistability is represented by region II with two stable steady states (a low [BDNF] state and a high [BDNF] state) along with an unstable steady state, while monostability with either low or high [BDNF] state is indicated by region I or III, respectively. Hence, with a starting point in region II, a pulse increase in *C*_post_ was able to trigger the transition of the BDNF concentration from a low to a high-level steady state, and the high-level state persisted even when *C*_post_ regained its low-level value (Figure 7B).

The premise of the realization of LTP at the stimulated synapse is that the cleft BDNF at the synapse can arrive at a high level after stimulation. Therefore, we predicted that to achieve the induction of LTP at a synapse, three steps had to be fulfilled as follows. Firstly, (*C*_post_, *m*_BDNF_) should lie in region I or II containing a low-level state such that the cleft BDNF concentration at the synapse [BDNF] is low prior to stimulation. Secondly, the synaptic stimulation prompts (*C*_post_, *m*_BDNF_) to enter region III at only a high state, and then [BDNF] switches from low to high. Thirdly, after stimulation, (*C*_post_, *m*_BDNF_) may remain in region III or II to maintain the high level [BDNF].

#### 3.2.2. Underlying mechanism of LTP induction

**Figure 8:**
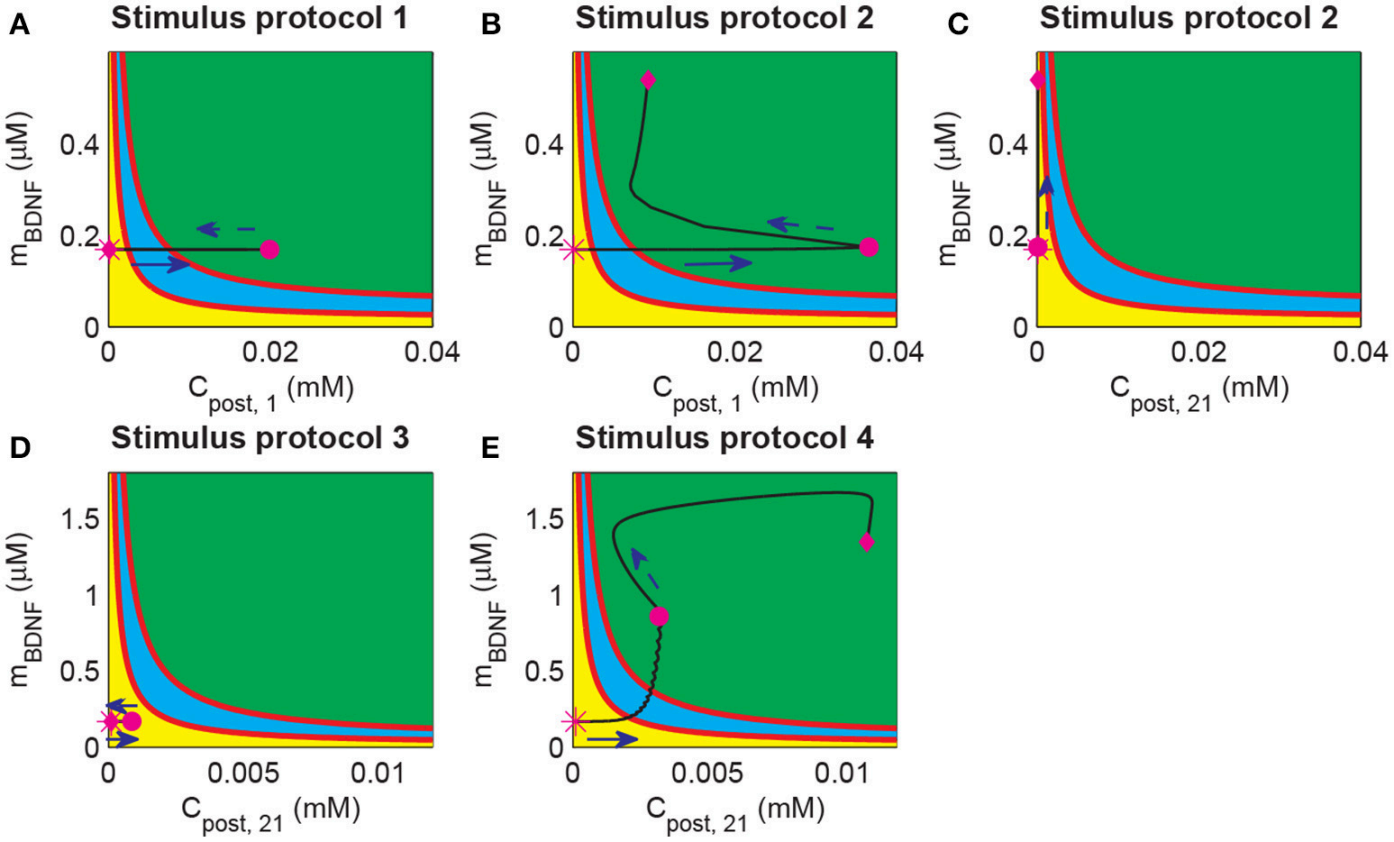
Projection of system trajectories onto the two-parameter bifurcation diagram of the local positive feedback loop model. **(A)** (*C*_post, 1_, *m*_BDNF_) lies in region I before and after stimulation (the protocol 1). **(B)** (*C*_post, 1_, *m*_BDNF_) lies in region I and region III before and after stimulation, respectively (the protocol 2). **(C)** (*C*_post, 21_, *m*_BDNF_) lies in region I before and after stimulation (the protocol 2). **(D)** (*C*_post, 21_, *m*_BDNF_) lies in region I before and after stimulation (the protocol 3). **(E)** (*C*_post, 21_, *m*_BDNF_) lies in region I and region III before and after stimulation (the protocol 4), respectively.

During the induction of LTP, it is essential to induce long-term plasticity via a transient stimulus. In previous simulations, a strong pulse in *C*_post_ occurred prior to the BDNF elevation and the increasing EPSP and EPSC amplitudes (Figure 4). These results suggested that the dynamics of *C*_post_ and BDNF transcription were important for LTP induction. To investigate the underlying mechanism of LTP induction based on the above bifurcation analysis of the toy model, we projected the trajectories of synapse 1 or 21 obtained from the whole system onto the two-parameter bifurcation diagram of the toy model (Figure 8). Here, we note that the postsynaptic Ca^2+^ (*C*_post_) was different among synapses, while the BDNF mRNA level (*m*_BDNF_) was global and had the same value for all synapses.

Before stimulation, the *C*_post_ and *m*_BDNF_ values for both synapses 1 and 21 were the same and located at region I (Figure 8, magenta stars). Under protocol 1, a 100 Hz stimulus was applied to synapse 1 in P1, which led to an elevation of *C*_post, 1_ but had no effect on *m*_BDNF_. Therefore, (*C*_post, 1_, *m*_BDNF_) reached region III at the end of the stimulation (magenta dot in Figure 8A). However, after withdrawal of the stimulation, *C*_post, 1_ decreased to its basal level and (*C*_post, 1_, *m*_BDNF_) returned to the low-level state at region I (magenta diamond in Figure 8A).

In protocol 2, many synapses in P1 were activated, and (*C*_post, 1_, *m*_BDNF_) reached the high-level state region III with a much higher level *C*_post_ at the end of the stimulation (Figure 8B, magenta dot). After stimulation, *C*_post_ decreased, but BDNF transcription was induced such that *m*_BDNF_ increasing, and in turn maintained the high level postsynaptic Ca^2+^ through the internal calcium release due to TrkB activation. In addition, (*C*_post, 1_, *m*_BDNF_) moved to and persisted at a high-level state at region III (Figure 8, magenta diamond). Hence, the cleft BDNF concentration at synapse 1 persisted at a high level, and LTP was induced by many synapses that were activated in P1, thus illustrating the cooperativity of LTP. Nevertheless, at unstimulated synapse 21 in P2, the postsynaptic Ca^2+^ concentration (*C*_post, 21_) showed little change, and (*C*_post, 21_, *m*_BDNF_) remained in the low state region I (Figure 8C). Consequently, the cleft BDNF concentration at synapse 21 remained low, and LTP did not occur at the synapses in P2. Thus, under protocol 2, LTP was induced in P1 with strong stimulation but not in P2, indicating the input specificity of LTP.

In the case of protocols 3 and 4, weak stimulation to P2 alone (protocol 3) could only trigger a brief increase in *C*_post, 21_, which returned to the basal value soon after the stimulation (see Figure 8D); however, when a weak stimulation to P2 was paired with a strong stimulation to P1 (protocol 4), (*C*_post, 21_, *m*_BDNF_) increased to the high state at region III (Figure 8E). Under protocol 3, (*C*_post, 21_, *m*_BDNF_) reached the magenta dot immediately due to the increase in both *C*_post_ and *m*_BDNF_. The increase in *m*_BDNF_ originated from the induction of BDNF transcription through CREB, which was activated by the intracellular Ca^2+^ signal (Figure 1). Next, internal calcium release was induced by TrkB activated by cleft BDNF, and (*C*_post, 21_, *m*_BDNF_) persisted at the high-level state (Figure 8E, magenta diamond). Hence, the results based on protocols 3 and 4 showed the associativity of LTP, in which a strong stimulus could pair with a weak stimulus and induce LTP at synapses with a weak stimulus.

The above findings revealed that the bistability of the local positive feedback motif of BDNF production could provide a unified mechanism of the cooperativity, input specificity, and associativity of LTP based on the dynamics of postsynaptic Ca^2+^ and BDNF transcription in response to the stimulus. Hence, the proposed toy model is able to predict the behaviors of the whole system and supply guidance for the realization of LTP.

### 3.3. LTP induction requires presynaptic neurotransmitter release and postsynaptic depolarization

In the above simulations, we explored the long-term behaviors in the postsynaptic neuron after stimulation, including changes in molecular concentrations, AMPA channel conductance, and EPSC and EPSP amplitudes. Next, we focus on the postsynaptic neuron activity during stimulation and discuss the role of the pre- and postsynaptic components in the realization of LTP.

**Figure 9:**
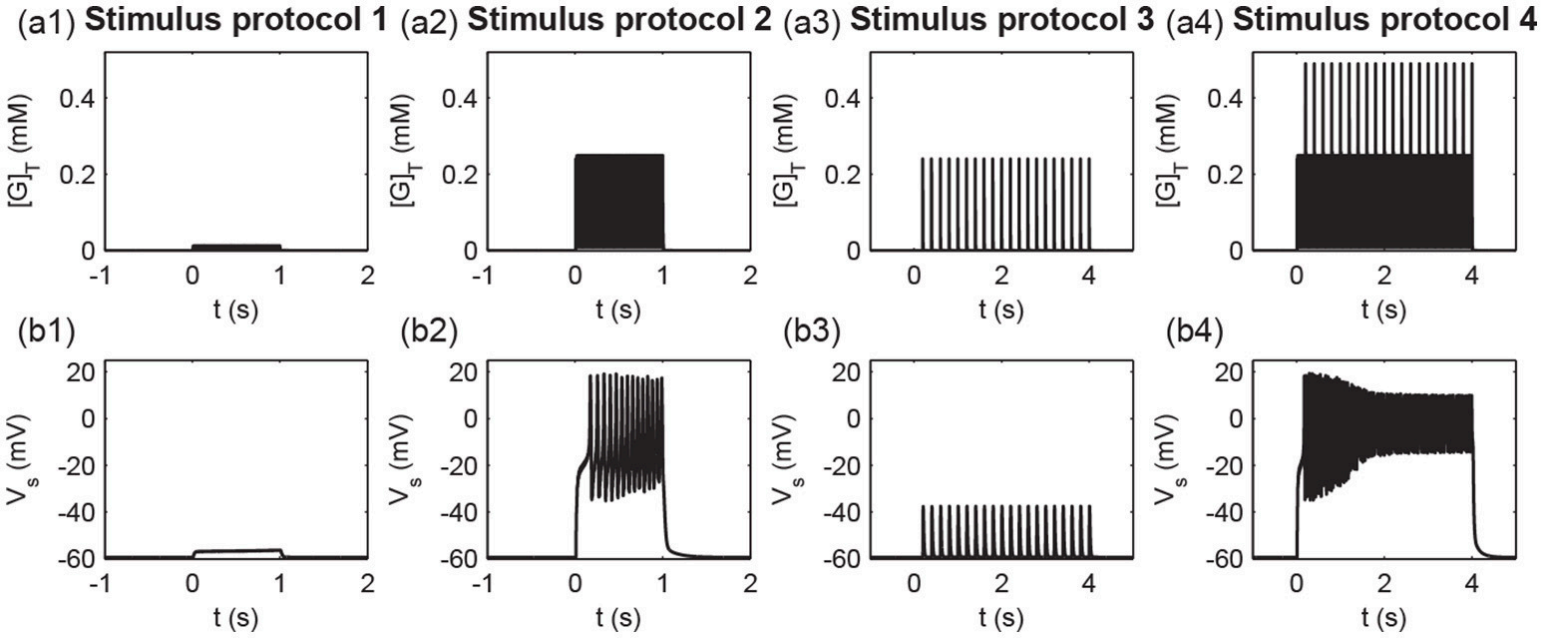
Times course of **(a1–a4)** the total glutamate concentration [G]_*T*_ and **(b1–b4)** the postsynaptic membrane potential *V*_*s*_ under the four stimulus protocols.

To measure different protocols quantitatively, we defined the total glutamate concentrations in the clefts released from all stimulated synapses [G]_*T*_ (${\left[\text{G}\right]}_{T}=\sum_{j=1}^{n}{\left[\text{G}\right]}_{j}$). Figure 9 shows the time courses of the total glutamate concentration and postsynaptic membrane potential *V*_*s*_ for the four stimuli protocols. As shown in Figure 9, weak inputs in protocols 1 and 3 induced small [G]_T_ and hence failed to elicit a postsynaptic action potential (Figure 9). Strong inputs in protocols 2 and 4 resulted in large [G]_T_ and caused the postsynaptic neuron to fire a burst of action potentials (Figure 9).

**Figure 10:**
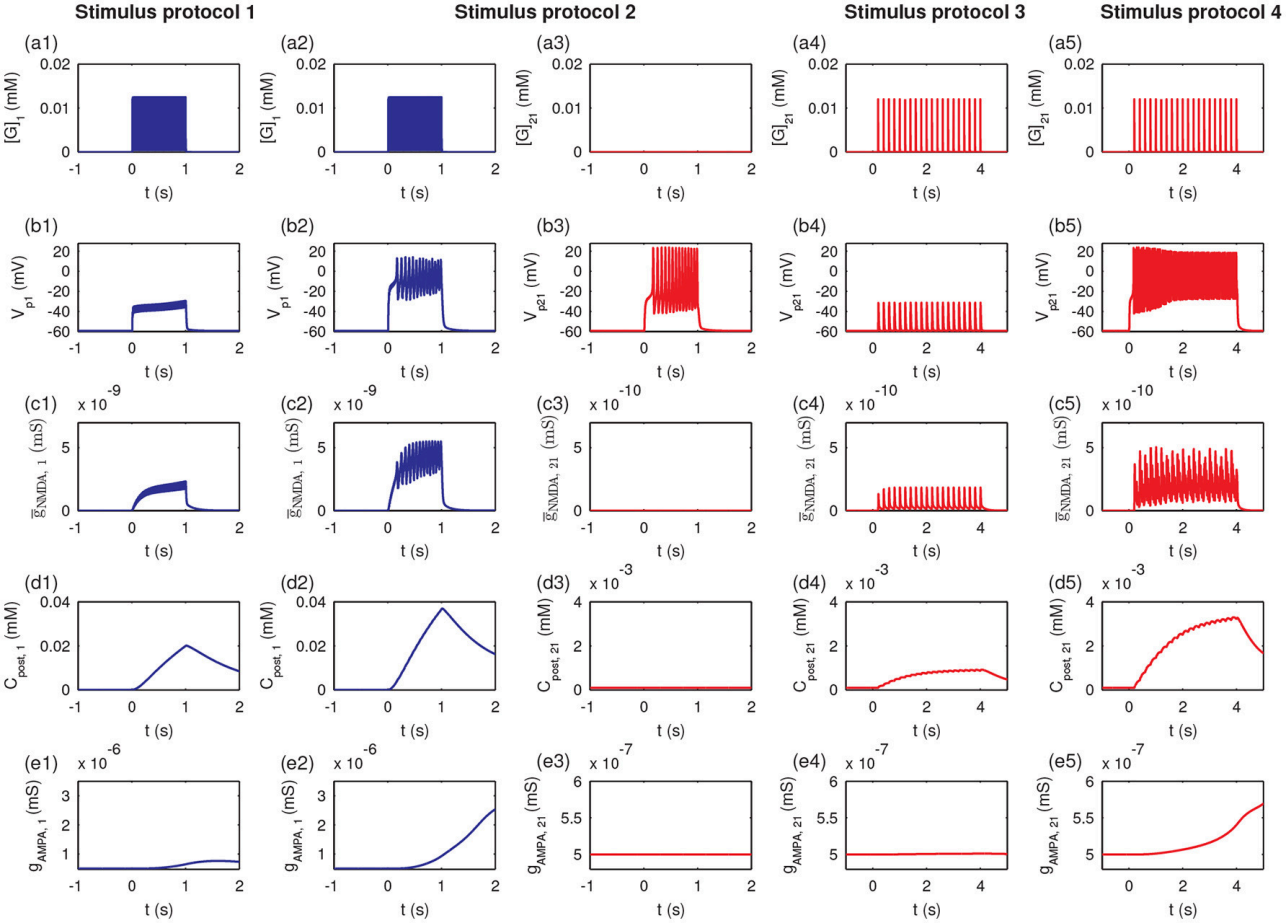
The induction of LTP requires presynaptic neurotransmitter release and postsynaptic action potentials. Figures show the time courses of the glutamate concentration in a cleft ([G]_*j*_), postsynaptic membrane potential (*V*_p*j*_), the conductance of the NMDA channels (ḡ_NMDA, *j*_), postsynaptic calcium concentrations (*C*_post, *j*_), and maximal conductance of AMPA receptor channels (*g*_AMPA, *j*_) for synapse *j*, respectively: **(a1–e1)** synapse 1 under protocol 1; **(a2–e2)** synapse 1 under protocol 2; **(a3–e3)** synapse 21 under protocol 2; **(a4–e4)** synapse 21 under protocol 2; **(a5–e5)** synapse 21 under protocol 4. In all cases, the stimulations (four stimulus protocols) are given at *t* = 0.

To further examine the detailed dynamics of the P1 and P2 pathway synapses during stimulation, we studied the postsynaptic responses at synapse 1 and synapse 21. In the protocol 1, the glutamate concentration in the cleft of synapse 1 ([G]_1_) rose due to the high-frequency synaptic transmission at synapse 1 (Figure 10a1); the postsynaptic membrane potential at synapse 1 (*V*_p1_) only exhibited a subthreshold oscillation (Figure 10b1). Thus, the conductance of the NMDA channels (ḡ_NMDA, 1_) only exhibited small changes (Figure 10c1) due to the voltage-dependent block of the NMDA channel by Mg^2+^. The postsynaptic Ca^2+^ influx was small (Figure 10d1), and the maximal conductance of AMPA receptor channels (*g*_AMPA, 1_) remained at a low level (Figure 10e1). In protocol 2, when all synapses in P1 were simultaneously activated, the glutamate concentration in the synapse 1 cleft rose (Figure 10a2), and the postsynaptic membrane fired action potentials (Figure 10b2). Then, the neurotransmitter binding paired with the postsynaptic depolarization to enhance the opening of the NMDA receptor channels and elevate NMDA channel conductance (Figure 10c2), resulting in a large Ca^2+^ influx (Figure 10d2) and consequent increase in the conductance of AMPA receptor channels (Figure 10e2). Therefore, protocol 1 with a stimulation of a single synapse could not elicit action potentials and high postsynaptic Ca^2+^ concentrations, while protocol 2 with stimulation of multiple synapses could induce high postsynaptic Ca^2+^ level, which is the basis for the cooperativity of LTP.

Under protocol 2, the glutamate concentration in the cleft of synapse 21 remained at a low level (Figure 10a3), and the postsynaptic action potential (Figure 10b3) was unable to elicit any change in NMDA channel conductance (Figure 10c3), the postsynaptic Ca^2+^ concentration (Figure 10d3), or the AMPA receptor channel conductance (Figure 10e3) at synapse 21; because no presynaptic neurotransmitter was released in P2 despite the postsynaptic membrane depolarization. Hence, LTP was induced only at synapses with stimulation (P1 pathway), and did not occur at other synapses onto the same neuron without stimulation (P2 pathway), which elucidates the input specificity of LTP.

Under protocol 3 with a weak stimulus to the P2 pathway, the presynaptic vesicle released with low frequency (Figure 10a4) resulted in subthreshold responses in the postsynaptic potential *V*_p21_ (Figure 10b4). Thus, small amplitude oscillations of the NMDA channel conductance (ḡ_NMDA, 21_ (Figure 10c4) and a minor increase in the postsynaptic calcium concentration *C*_post, 21_ occurred (Figure 10d4). Hence, the weak stimulus failed to induce an elevation of the maximal conductance of AMPA receptor channels at synapse 21 (Figure 10e4). When the weak stimulus to P2 was paired with the strong stimulus to P1 (protocol 4), despite the low frequency presynaptic vesicle release at synapse 21 (Figure 10a5), the postsynaptic membrane of synapse 21 could be depolarized with an obvious action potential (Figure 10b5). The high frequency action potential led to a clear increase in NMDA channel conductance (Figure 10c5) and a rise in the postsynaptic calcium concentration (Figure 10d5). The high level postsynaptic calcium eventually elevated the AMPA receptor channel conductance at synapse 21 to induce LTP (see Figure 10e5). These results demonstrated that the strong stimulation in the P1 pathway could lead to the depolarization of the postsynaptic neurons at synapses in the P2 pathway with weak stimulation, revealing the associativity of LTP.

**Figure 11:**
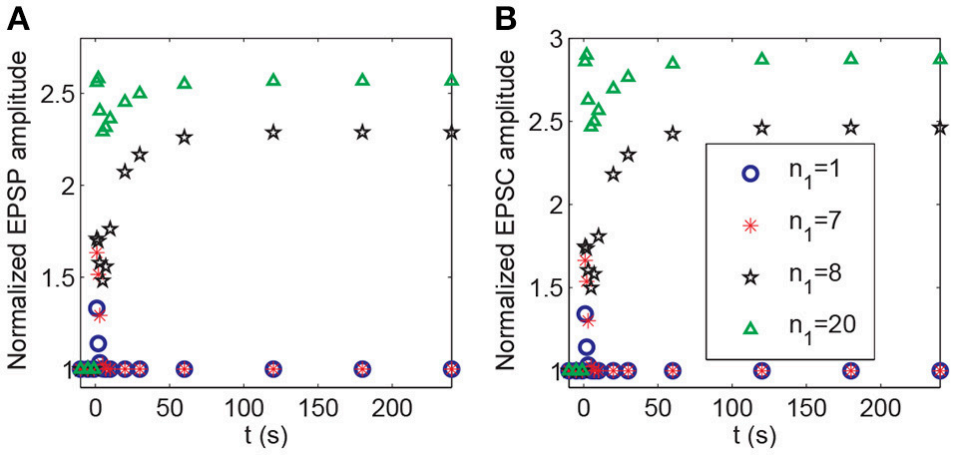
Dependence of LTP induction on the number of active synapses in pathway P1. Time courses of **(A)** the normalized amplitudes of EPSP and **(B)** AMPA-mediated EPSC of synapse 1 after stimulation (the protocol 2) for different values of *n*_1_. The stimulations are given at *t* = 0.

In the above demonstration of cooperativity, LTP in synapse 1 could not be induced by a stimulus to only synapse 1; however, it could be induced when *n*_1_ = 20 synapses in the P1 pathway were subjected to the stimulus. Hence, the number of stimulated synapses might affect the induction of LTP. Thus, we altered the synapse number *n*_1_ in the P1 pathway (and set *n*_2_ = *n*−*n*_1_) and applied the protocol 2. Our simulation showed a threshold response with respect to changes in the number *n*_1_. LTP at synapse 1 could not be induced at synapse 1 when *n*_1_ ≤ 7 and was induced when *n*_1_≥8, as shown by the clear increase in the normalized amplitudes of both EPSP and AMPA-mediated EPSC (Figure 11).

**Figure 12:**
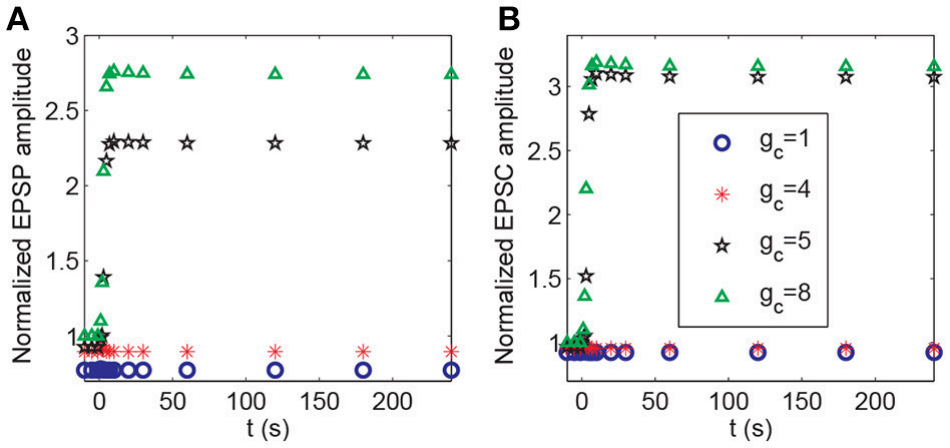
Dependence of LTP induction on the coupling conductance between the soma and the spine membrane potentials. Time courses of **(A)** the normalized amplitudes of EPSP and **(B)** AMPA-mediated EPSC of synapse 21 after stimulation (the protocol 4) for different values of *g*_c_. The stimulations are given at *t* = 0.

During the associativity of LTP, a strong stimulus to P1 can evoke LTP at P2 synapses under weak stimulation, suggesting that the input signal to P1 synapses can be propagated to P2 synapses. In the above analysis, we demonstrated that BDNF transcription represented a global effect connecting P1 and P2 synapses (Figure 8). In addition, depolarization of the postsynaptic membrane at synapses in P2 was associated with action potentials of the pyramidal neuron, which was affected by the strong stimulation of P1. Thus, the propagation between the membrane potential of the soma and spines could be essential for inducing LTP at P2. In the model, the efficiency of coupling between the soma and spine membrane potentials was represented by the coefficient *g*_c_ in Equation A2 (Appendix in Supplementary Material). We varied the parameter *g*_c_ and applied protocol 4 to examine how it might affect the responses in synapse 21. The results showed a threshold response with respect to changes in *g*_c_. From Figure 12, LTP in synapse 21 was not induced when $g_{\text{c}}\leq 4\text{ mS}/\text{c}{\text{m}}^{2}$, and when $g_{\text{c}}\geq 5\text{ mS}/\text{c}{\text{m}}^{2}$, the normalized amplitudes of EPSP and AMPA-mediated EPSC in P2 rapidly increased from low to high after stimulation, indicating an induction of LTP at the P2 synapse.

## Discussion

Long-term potentiation has three essential properties: input specificity, cooperativity, and associativity. We have developed a computational model based on the molecular processes of synaptic plasticity to discover the unified mechanisms of the three properties of LTP. The model integrates gene expression regulation with neuronal activity of a hippocampal pyramidal neuron with multiple excitatory synapses. The model is able to realize the three properties of LTP which are measured by changes in the amplitudes of EPSP and AMPA-mediated EPSC in response to different stimulus protocols (Figure 3). Model simulations showed that the local synthesis of BDNF proteins at each synapse is important for the induction of LTP. We proposed a toy model of local BDNF dynamics based on the positive feedback motif of postsynaptic BDNF translation and TrkB receptor activation (Figure 5). Bifurcation analysis of the toy model revealed a unified mechanism of the three properties of LTP, which was illustrated by the bistability of the positive feedback motif and the dynamic responses of the postsynaptic calcium concentration and BDNF mRNA to various stimulus protocols (Figure 8).

In our model, the induction of LTP requires both presynaptic and postsynaptic activity since both binding of the neurotransmitter and depolarization of the postsynaptic membrane are required for the opening of NMDA channels. The cooperation between activated synapses evokes the switches from low to high level presynaptic vesicle release and the postsynaptic action potential to induce LTP through a bistable regulation of BDNF activity (Figure 11). Moreover, propagation of the action potential through the coupling between the membrane potentials of soma and spines plays important roles in the associativity of LTP (Figure 12). Through the propagation of action potentials, the strong stimulus to the pathway P1 can induce LTP in the pathway P2 with a weak stimulus.

Based on the study, we conclude that the mechanisms involved in the induction of LTP include the following:

1. Positive feedback regulation of local BDNF protein synthesis such that bistable BDNF activity in each synapse is possible via the regulation of the postsynaptic calcium concentration or BDNF transcription.
2. Sufficiently strong neurotransmitter release induced by the cooperation synapse during stimulation.
3. Propagation of action potentials through the connection between membrane potentials of soma and spines, such that a synapse with a weak stimulus can be depolarized by synapses with a strong stimulus.

These mechanisms together explain the input specificity, cooperativity, and associativity of LTP.

Existing models (Kitajima and Hara, 1991; Migliore et al., 1997) only consider phosphorylation processes, and do not involve transcriptional regulations that are essential for long-lasting forms of synaptic plasticity. The unified mechanism responsible for the cooperativity, input specificity and associativity of LTP has not been developed in these models. The model proposed in this paper can achieve the three properties of LTP by including more detailed molecular processes of transcription and local translation. These extensions enable us to construct a more biophysically reasonable model and to discover additional results about the molecular mechanisms not shown in previous models.

Associative LTP, like associative Pavlovian conditioning, links an event (a conditioned stimulus, CS) with another event (an unconditioned stimulus, US) (Kandel et al., 2013), is closely related to the process of learning and memory. The process of LTP is an important mechanism of memory storage in the hippocampal system and contributes to the associative classical conditioning in the amygdala. According to the cellular hypothesis of cued fear conditioning, the strength of synapses that transmit CS information to principal neurons in the lateral amygdala (LA) increases when the CS is paired with US, and this associative LTP may also be a mechanism for storing memories of the CS-US association (Blair et al., 2001). The expression of BDNF during a stimulus has been implicated in fear memory consolidation (Johansen et al., 2011). The proposed model in this study can also be applied for the development of computational models to investigate the mechanism of fear memory.

The proposed computational model expounds the dynamic mechanisms of excitatory synapses underlying the three important properties of LTP. The model also provides a module that can be used in the biophysical modeling of neural networks for synaptic plasticity, learning and memory. In this model, regulation through local mRNA translation and BDNF transcription is essential for the maintenance of LTP. Unlike LTP, the regulation of gene expression is not involved in short-term potentiation (STP). Hence, as a model of synapse dynamics, modifications of the proposed model with further details of STP could be capable of dealing with both the induction and maintenance of synaptic plasticity. In our model, to achieve long-term potentiation of the synaptic transmission, we introduced a positive feedback loop in each synapse that produces bistability. However, after the induction of LTP, the synaptic efficacy might not return to the low state if it remains in the high state for a long time, which is not completely in agreement with the experiments. To return to the low state, one might consider some other biological processes, such as degeneration of neurons, random inactivation of gene expressions, or changes in epigenetic states, etc. With the accumulation of experimental results concerning complex reactions that are localized to the postsynaptic density, it is feasible to add these details to the model. For future studies, computational models that involve multiple-level processes, including neural circuits, synapses and more detailed biochemical reactions, are required to facilitate the establishment of a more complete model to understand the synaptic plasticity and facilitate learning and memory.

## Author contributions

LH, ZY, and JL designed and performed the research and wrote the article.

### Conflict of interest statement

The authors declare that the research was conducted in the absence of any commercial or financial relationships that could be construed as a potential conflict of interest.
