# Supplementary material for: Underlying Mechanisms of Cooperativity, Input Specificity, and Associativity of Long-Term Potentiation Through a Positive Feedback of Local Protein Synthesis
Source: Front Comput Neurosci. 2018 May 1;12:25. doi: 10.3389/fncom.2018.00025 (PMC5938377; doi:10.3389/fncom.2018.00025)
Supplement: Supplementary file 1 [file Presentation_1.pdf]

APPENDIX: Underlying Mechanisms of LTP

April 16, 2018

Formulations of the Model

In the model formulations, we considered the dynamics of postsynaptic membrane potential based on the Morris-Lecar model (Morris and Lecar, 1981). Construction of the multi-compartment pyramidal cell model is inspired by previous multi-compartment neuron models (Traub et al., 1991; Pinsky and Rinzel, 1994; Li et al., 2009). All compartments have the following currents: leak (I_{L}), potassium (I_{K}) and calcium (I_{Ca}) (Morris and Lecar, 1981). In addition, the spines have an AMPA current (I_{AMPA}) and an NMDA current (I_{NMDA}). Upon stimulation, the presynaptic neuron releases neurotransmitter to trigger the postsynaptic membrane potential.

First, we referred to the discrete vesicle release model for the neurotransmitter dynamics in each synapse (Tsodyks and Markram, 1997; Nadkarni et al., 2008). There are n $(j = 1, 2, \cdots, n)$ synapses in the dendrite (Fig. 1A). For each synapse, the changes of glutamate concentration $[\mathrm{G}]_j$ in the cleft is given by the equation

$$\frac{d[\mathrm{G}]_j}{dt} = -\frac{[\mathrm{G}]_j}{\tau_{\mathrm{in}}} + \delta(t - t_{i,j})u, \tag{A1}$$

where τ_{in} is the time constant of inactivation. Here, the delta function $\delta(t - t_{i,j})$ represents the events of stimulated vesicle release at each synapse at the discrete time series $t_{i,j}$. The time series $t_{i,j}$ is determined by the protocol of the stimulus to the specific synapse. In this study, we divided the synapses into two pathways, which include n_1 (the pathway P1, synapses $j = 1, \cdots, n_1$) and n_2 (the pathway P2, synapses $j = n_1 + 1, \cdots, n_1 + n_2 = n$) synapses, respectively, and different stimulus protocols are applied to the two pathways. In each synapse, the

neurotransmitter content of a vesicle, u, is released into the synaptic cleft upon vesicle release.

We assumed that the area of a single spine is A, and the equations for the membrane potential at each compartment of the postsynaptic neuron are formulated as

$$\begin{aligned}
C_m \frac{dV_{\mathrm{s}}}{dt} &= -g_{\mathrm{L}}(V_{\mathrm{s}} - V_{\mathrm{L}}) - \sum_{j=1}^{n} g_{\mathrm{c}}(V_{\mathrm{s}} - V_{\mathrm{p}j}) && \text{(A2)} \\
&\quad - g_{\mathrm{Ca}} m_{\mathrm{s},\infty}(V_{\mathrm{s}} - V_{\mathrm{Ca}}) - g_{\mathrm{K}} n_{\mathrm{s}}(V_{\mathrm{s}} - V_{\mathrm{K}}) + I_0, \\
AC_m \frac{dV_{\mathrm{p}j}}{dt} &= -Ag_{\mathrm{L}}(V_{\mathrm{p}j} - V_{\mathrm{L}}) - Ag_{\mathrm{c}}(V_{\mathrm{p}j} - V_{\mathrm{s}}) && \text{(A3)} \\
&\quad - Ag_{\mathrm{Ca}} m_{\mathrm{p}j,\infty}(V_{\mathrm{p}j} - V_{\mathrm{Ca}}) - Ag_{\mathrm{K}} n_{\mathrm{p}j}(V_{\mathrm{p}j} - V_{\mathrm{K}}) \\
&\quad - I_{\mathrm{AMPA},j} - I_{\mathrm{NMDA},j}, \\
\frac{dn_{\mathrm{s}}}{dt} &= (n_{\mathrm{s},\infty} - n_{\mathrm{s}})/\tau_{n,\mathrm{s}}, && \text{(A4)} \\
\frac{dn_{\mathrm{p}j}}{dt} &= (n_{\mathrm{p}j,\infty} - n_{\mathrm{p}j})/\tau_{n,\mathrm{p}j}, && \text{(A5)} \\
\frac{dr_{a,j}}{dt} &= \alpha_{\mathrm{AMPA}}[\mathrm{G}]_j(1 - r_{a,j}) - \beta_{\mathrm{AMPA}} r_{a,j}, && \text{(A6)} \\
\frac{dr_{n,j}}{dt} &= \alpha_{\mathrm{NMDA}}[\mathrm{G}]_j(1 - r_{n,j}) - \beta_{\mathrm{NMDA}} r_{n,j}. && \text{(A7)}
\end{aligned}$$

Here, V_{s} and $V_{\mathrm{p}j}$ are membrane potentials at the soma and spines, respectively. The equations (A2)-(A3) refer to the Morris-Lecar model in which calcium and potassium currents are considered, and the postsynaptic currents are mainly involved in the dynamics of the membrane potentials at spines. In (A2), g_c is the coupling conductance between the soma and the spine, and I_0 represents the integration of all possible external stimulations other than from synapses 1 to n onto the pyramidal neuron. The potassium currents are described by the ion channel opening rates n_s and $n_{\mathrm{p}j}$ through the equations (A4) and (A5); the opening rate of the calcium ion channel is assumed to be a constant $m_{s,\infty}$. The gating variables $m_{k,\infty}$, $n_{k,\infty}$ and $\tau_{n,k}$ ($k =$s or pj ($j = 1, 2, \cdots, n$)) are (Morris and Lecar, 1981):

$$\begin{aligned}
m_{k,\infty} &= \frac{1}{2}(1 + \tanh((V_k - v_1)/v_2)), && \text{(A8)} \\
n_{k,\infty} &= \frac{1}{2}(1 + \tanh((V_k - v_3)/v_4)), && \text{(A9)} \\
\tau_{n,k} &= \frac{1}{\phi \cosh((V_k - v_3)/(2v_4))}. && \text{(A10)}
\end{aligned}$$

In the model, AMPA and NMDA receptors are placed in the dendritic spines. The excitatory postsynaptic currents mediated by the AMPA and NMDA channels at each spine are given by

$$I_{\mathrm{AMPA},j} = \bar{g}_{\mathrm{AMPA},j}(V_{\mathrm{p}j} - V_E), \tag{A11}$$
$$I_{\mathrm{NMDA},j} = \bar{g}_{\mathrm{NMDA},j}(V_{\mathrm{p}j} - V_E), \tag{A12}$$

where $\bar{g}_{\mathrm{AMPA},j}$ and $\bar{g}_{\mathrm{NMDA},j}$ are conductances of the AMPA and NMDA channels at each spine, which are denoted as

$$\bar{g}_{\mathrm{AMPA},j} = g_{\mathrm{AMPA},j} r_{a,j}, \tag{A13}$$
$$\bar{g}_{\mathrm{NMDA},j} = \frac{g_{\mathrm{NMDA},j} r_{n,j}}{1 + [\mathrm{Mg}^{2+}] \exp(-0.062 V_{\mathrm{p}j})/3.57}. \tag{A14}$$

Here, $g_{\mathrm{AMPA},j}$ is the maximal conductance of the AMPA channel at each spine that is dependent on BDNF activity and given by (A15) below, and $r_{a,j}$ and $r_{n,j}$ are fractions of the AMPA and NMDA receptors in the open state and are provided by (A6)-(A7). In (A6), α_{AMPA} and β_{AMPA} are the opening and closing rates of the AMPA receptor, respectively. Similarly, $g_{\mathrm{NMDA},j}$, $r_{n,j}$, α_{NMDA} and β_{NMDA} are corresponding variables for the NMDA channel, and the equation for $g_{\mathrm{NMDA},j}$ is given by (A16) below. The AMPAR and NMDAR equilibrium potentials are assumed to be the same value V_E (Destexhe et al., 1998). Moreover, the NMDA receptor channels are blocked by Mg^{2+} in a voltage-dependent manner (Jahr and Stevens, 1991), and the NMDA current is dependent on the concentration of extracellular magnesium ($[\mathrm{Mg}^{2+}]$).

Postsynaptic Ca^{2+} can either increase AMPA channel conductance by promoting GluR1 insertion or decrease in AMPA channel conductance by inducing the internalization of AMPA receptors. Moreover, BDNF regulates AMPA receptor subunit expression and induces the delivery of AMPA receptors to the synapse. Therefore, we assumed that the maximal conductance of the AMPA channel was dependent on the postsynaptic calcium concentration ($C_{\mathrm{post},j}$) and the concentration of BDNF in the synaptic cleft ($[\mathrm{BDNF}]_j$). The dependence on postsynaptic Ca^{2+} for the increase and decrease in AMPA channel maximal conductance were formulated as Michaelis-Menten functions

$$\frac{dg_{\mathrm{AMPA},j}}{dt} = k_{bA} + k_{gA} \frac{C_{\mathrm{post,j}}}{K_{gA} + C_{\mathrm{post,j}}} \frac{[\mathrm{BDNF}]_j}{K_{BA} + [\mathrm{BDNF}]_j} - k_A \frac{C_{\mathrm{post,j}}}{K_A + C_{\mathrm{post,j}}} g_{\mathrm{AMPA},j} - k_{dA} g_{\mathrm{AMPA},j}, \tag{A15}$$

where k_{bA} and k_{dA} are the basal rate of the increase and the rate of decrease of AMPA channel maximal conductance, respectively. Michaelis-Menten functions are often used in the context of enzyme kinetics (Alon, 2006).The concentration of CaMKII subunits in the postsynaptic density (PSD) is high, while the concentration of PP1 is much lower (Lisman and Zhabotinsky, 2001). Therefore, $K_{gA} > K_A$ and $k_{gA} > k_A$.

The maximal NMDA conductance $g_{\mathrm{NMDA},j}$ is dependent on the extracellular BDNF concentration ($[\mathrm{BDNF}]_j$) as a result of TrkB activation following BDNF binding and was modeled according to Michaelis-Menten dynamics:

$$\frac{dg_{\mathrm{NMDA},j}}{dt} = k_{bN} + k_{gN}\frac{[\mathrm{BDNF}]_j}{K_{BN} + [\mathrm{BDNF}]_j} - k_{dN}g_{\mathrm{NMDA},j}, \quad \text{(A16)}$$

where k_{bN} and k_{dN} are basal rates of increasing and decreasing NMDA channel maximal conductance, respectively.

The dynamics of postsynaptic BDNF is regulated by the activation of CREB and calcium signaling. Active CREB upregulates the transcription of postsynaptic BDNF ($[\mathrm{BDNF}]_{\mathrm{post},j}$ for the concentration), and the activation of CREB is dependent on the intracellular signaling pathways that involve intracellular Ca^{2+} and extracellular BDNF through the TrkB receptor. Therefore, the intracellular BDNF transcription rate is provided as a function of [CREB] ($k_B([\mathrm{CREB}])$); the CREB activation rate is expressed as a function $k_b(C_\mathrm{T}, [\mathrm{BDNF}]_\mathrm{T})$ of the total postsynaptic calcium and synaptic cleft BDNF concentrations C_T and $[\mathrm{BDNF}]_\mathrm{T}$ ($C_\mathrm{T} = \sum_{j=1}^{n} C_{\mathrm{post},j}$, $[\mathrm{BDNF}]_\mathrm{T} = \sum_{j=1}^{n}[\mathrm{BDNF}]_j$). Extracellular BDNF can induce mTOR-dependent local protein synthesis in dendrites; thus, we assumed that the translation of BDNF mRNA (m_{BDNF} for the concentration) in each dendritic spine was regulated by the level of BDNF in the synaptic cleft; the translation rate at the j'th spine is given by a function $k_{p,j}(m_{\mathrm{BDNF}}, [\mathrm{BDNF}]_j)$ defined below. In this work, we only considered the secretion of BDNF from spine and the endocytic uptake of BDNF into the postsynaptic dendritic spines for the exchanges of BDNF proteins between cellular and synaptic cleft. We did not distinguish between post-Golgi granules and endocytosed BDNF-containing endosomes in our model, and assumed that both endogenous BDNF and endocytosed BDNF were secreted depending on the postsynaptic Ca^{2+} level. These

processes led to the following equations:

$$\frac{d[\text{CREB}]}{dt} = k_b(C_\text{T}, [\text{BDNF}]_\text{T}) - k_{d,\text{CREB}}[\text{CREB}], \tag{A17}$$

$$\frac{dm_\text{BDNF}}{dt} = k_B([\text{CREB}]) - k_{dm}m_\text{BDNF}, \tag{A18}$$

$$\frac{d[\text{BDNF}]_{\text{post},j}}{dt} = k_{p,j}(m_\text{BDNF}, [\text{BDNF}]_j) + k_\text{in}[\text{BDNF}]_j - k_\text{out}C_{\text{post},j}[\text{BDNF}]_{\text{post},j} - k_{dB}[\text{BDNF}]_{\text{post},j}, \tag{A19}$$

$$\frac{d[\text{BDNF}]_j}{dt} = k_\text{out}C_{\text{post},j}[\text{BDNF}]_{\text{post},j} - k_\text{in}[\text{BDNF}]_j - k_{dB}[\text{BDNF}]_j, \tag{A20}$$

where $k_{d,\text{CREB}}$ is the inactivation rate of CREB, k_in and k_out are the endocytosis and secretion rates of BDNF, and k_{dm} and k_{dB} are the degradation rates of BDNF mRNA and BDNF protein, respectively.

Michaelis-Menten kinetics is often used to non-cooperative biochemical reactions (Chen et al., 2010), and Hill type functions are often used in modeling cooperative biological processes (Gesztelyi et al., 2012; Stefan and Le Novère, 2013). In equations (A17)-(A20), the translation rate $k_{p,j}(m_\text{BDNF}, [\text{BDNF}]_j)$ at each spine is given by a Hill type function (Alon, 2006):

$$k_{p,j}(m_\text{BDNF}, [\text{BDNF}]_j) = k_1 m_\text{BDNF} + k_2 m_\text{BDNF}\frac{[\text{BDNF}]_j^2}{K_2^2 + [\text{BDNF}]_j^2}, \tag{A21}$$

where k_1 is the basal translation rate independent of the cleft BDNF. The BDNF transcription rate $k_B([\text{CREB}])$ is formulated as a Hill type function:

$$k_B([\text{CREB}]) = k_{bm} + k_B\frac{[\text{CREB}]^2}{K_B^2 + [\text{CREB}]^2}, \tag{A22}$$

where k_{bm} represents the basal transcription rate. The CREB activation rate $k_b(C_\text{T}, [\text{BDNF}]_\text{T})$ is provided by

$$k_b(C_\text{T}, [\text{BDNF}]_\text{T}) = k_{b,\text{CREB}} + k_C C_\text{T} + k_{BT}\frac{[\text{BDNF}]_\text{T}}{K_{BT} + [\text{BDNF}]_\text{T}}, \tag{A23}$$

where $k_{b,\text{CREB}}$ is the basal activation rate, and the dependence on $[\text{BDNF}]_\text{T}$ is provided by the Michaelis-Menten function.

The postsynaptic Ca^{2+} released from the internal calcium stores include the basal release (with a rate $k_{b,\text{post}}$) and the release induced by TrkB activation

(with a rate $k_{\text{post}}([\text{BDNF}]_j)$) when postsynaptic TrkB receptors are bound to the cleft BDNF. In addition, the sources of postsynaptic Ca^{2+} include an influx through NMDARs (with a rate $k_N \bar{g}_{\text{NMDA},j}(V_{\text{Ca}} - V_{\text{p}j})$). Thus, the dynamics of the postsynaptic Ca^{2+} concentrations at each synapse are formulated as

$$\frac{dC_{\text{post},j}}{dt} = k_{b,\text{post}} + k_N \bar{g}_{\text{NMDA},j}(V_{\text{Ca}} - V_{\text{p}j}) + k_{\text{post}}([\text{BDNF}]_j) - \frac{C_{\text{post},j}}{\tau_{\text{Ca}}}, \quad \text{(A24)}$$

where τ_{Ca} is the time constant of the Ca^{2+} clearance, and the rates of BDNF-induced postsynaptic Ca^{2+} release are given by a Michaelis-Menten function

$$k_{\text{post}}([\text{BDNF}]_j) = k_{\text{post}} \frac{[\text{BDNF}]_j}{K_{\text{post}} + [\text{BDNF}]_j}. \quad \text{(A25)}$$

Default parameter values used in our study are listed in Table A1. The parameters in the equations for the membrane potential are referred to the published literature (Morris and Lecar, 1981). Other parameters are calculated to realize the three properties of LTP.

References

Alon, U. (2006). *An introduction to system biology – Design principles of biological circuit* (Chapman & Hall/CRC, London)

Chen, W. W., Niepel, M., and Sorger, P. K. (2010). Classic and contemporary approaches to modeling biochemical reactions. *Genes Dev* 24, 1861–1875

Destexhe, A., Mainen, Z. F., and Sejnowski, T. J. (1998). *Kinetic models of synaptic transmission* (In: Koch, C., Segev, I. (eds.) Methods in Neuronal Modeling, MIT Press, Cambridge). 1–25

Gesztelyi, R., Zsuga, J., Kemeny-Beke, A., Varga, B., Juhasz, B., and Tosaki, A. (2012). The Hill equation and the origin of quantitative pharmacology. *Arch. Hist. Exact Sci.* 66, 427–438

Jahr, C. E. and Stevens, C. F. (1991). Voltage dependence of NMDA-activated macroscopic conductances predicted by single-channel kinetics. *J. Neurosci.* 10, 3178–3182

Table A1: Default parameter values of the model.

Parameter	Value	Parameter	Value
$C(\mu\mathrm{F/cm^2})$	20	$g_{\mathrm{L}}(\mathrm{mS/cm^2})$	2
$g_{\mathrm{Ca}}(\mathrm{mS/cm^2})$	4	$g_{\mathrm{K}}(\mathrm{mS/cm^2})$	8
$V_{\mathrm{L}}(\mathrm{mV})$	-60	$V_{\mathrm{Ca}}(\mathrm{mV})$	120
$V_{\mathrm{K}}(\mathrm{mV})$	-80	$v_1(\mathrm{mV})$	-1
$v_2(\mathrm{mV})$	15	$v_3(\mathrm{mV})$	10
$v_4(\mathrm{mV})$	14.5	$\phi(\mathrm{ms^{-1}})$	1/15
$g_c(\mathrm{mS/cm^2})$	8	$V_E(\mathrm{mV})$	0
$\tau_{\mathrm{in}}(\mathrm{ms})$	3	$u(\mathrm{mM})$	0.012
$\alpha_{\mathrm{AMPA}}((\mathrm{mMms})^{-1})$	1.1	$\beta_{\mathrm{AMPA}}(\mathrm{ms^{-1}})$	0.19
$\alpha_{\mathrm{NMDA}}((\mathrm{mMms})^{-1})$	0.072	$\beta_{\mathrm{NMDA}}(\mathrm{ms^{-1}})$	0.0066
$k_{bA}(\mathrm{mS/cm^2ms^{-1}})$	1×10^{-9}	$k_{dA}(\mathrm{ms^{-1}})$	0.002
$k_A(\mathrm{mS/cm^2ms^{-1}})$	1×10^{-8}	$K_A(\mathrm{mM})$	8×10^{-4}
$k_{gA}(\mathrm{mS/cm^2ms^{-1}})$	1×10^{-7}	$K_{gA}(\mathrm{mM})$	0.2
$K_{BA}(\mu\mathrm{M})$	1	$A(cm^2)$	2×10^{-9}
$k_{bN}(\mathrm{mS/cm^2ms^{-1}})$	5×10^{-10}	$k_{dN}(\mathrm{ms^{-1}})$	0.003
$k_{gN}(\mathrm{mS/cm^2ms^{-1}})$	1×10^{-9}	$K_{BN}(\mathrm{mM})$	50
$k_{b,\mathrm{CREB}}(\mu\mathrm{Mms^{-1}})$	8.0×10^{-6}	$k_{d,\mathrm{CREB}}(\mathrm{ms^{-1}})$	0.001
$k_{BT}(\mu\mathrm{Mms^{-1}})$	5.0×10^{-5}	$K_{BT}(\mu\mathrm{M})$	2
$k_C(10^{-3}\mathrm{ms^{-1}})$	5.0×10^{-4}	$\tau_{\mathrm{Ca}}(\mathrm{ms})$	1000
$k_{bm}(\mu\mathrm{Mms^{-1}})$	1.0×10^{-5}	$k_{dm}(\mathrm{ms^{-1}})$	6.0×10^{-5}
$k_B(\mu\mathrm{Mms^{-1}})$	0.001	$K_B(\mu\mathrm{M})$	1
$k_{dB}(\mathrm{ms^{-1}})$	0.001	$k_1(\mathrm{ms^{-1}})$	8.0×10^{-4}
$k_2(\mathrm{ms^{-1}})$	0.08	$K_2(\mu\mathrm{M})$	0.69
$k_{\mathrm{in}}(\mathrm{ms^{-1}})$	0.01	$k_{\mathrm{out}}((\mathrm{mMms})^{-1})$	0.5
$k_{b,\mathrm{post}}(\mathrm{mMms^{-1}})$	1.0×10^{-7}	$k_N(\mathrm{mMcm^2}(\mathrm{ms}\mu\mathrm{A})^{-1})$	120
$k_{\mathrm{post}}(\mathrm{mMms^{-1}})$	1.2×10^{-5}	$K_{\mathrm{post}}(\mu\mathrm{M})$	4
$[\mathrm{Mg^{2+}}](\mathrm{mM})$	1	$I_0(\mu\mathrm{A/cm^2})$	30

Li, G., Nair, S. S., and Quirk, G. J. (2009). A biologically realistic network model of acquisition and extinction of conditioned fear associations in lateral amygdala neurons. *J. Neurophysiol.* 101, 1629–1646

Lisman, J. E. and Zhabotinsky, A. M. (2001). A model of synaptic memory: a CaMKII/PP1 switch that potentiates transmission by organizing an AMPA receptor anchoring assembly. *Neuron* 31, 191–201

Morris, C. and Lecar, H. (1981). Voltage oscillations in the barnacle giant muscle fiber. *Biophys. J.* 35, 193–213

Nadkarni, S., Jung, P., and Levine, H. (2008). Astrocytes optimize the synaptic transmission of information. *PLoS Comput. Biol.* 4, e1000088

Pinsky, P. F. and Rinzel, J. (1994). Intrinsic and network rhythmogenesis in a reduced Traub model for CA3 neurons. *J. Comput. Neurosci.* 1, 39–60

Stefan, M. I. and Le Novère, N. (2013). Cooperative Binding. *PLoS Comput Biol* 9, e1003106–

Traub, R. D., Wong, R. K. S., Miles, R., and Michelson, H. (1991). A model of a CA3 hippocampal pyramidal neuron incorporating voltage-clamp data on intrinsic conductances. *J. Neurophysiol.* 66, 635–650

Tsodyks, M. V. and Markram, H. (1997). The neural code between neocortical pyramidal neurons depends on neurotransmitter release probability. *Proc. Natl. Acad. Sci. USA.* 94, 719–723
